# Defective Endothelial Glutaminolysis Contributes to Impaired Angiogenesis and Poor Ischemic Tissue Repair in Diabetes

**DOI:** 10.34133/research.0706

**Published:** 2025-05-22

**Authors:** Meina Zhao, Jiaheng Zhou, Yang Hu, Xinpei Wang, Jiong An, Meijie Liu, Pengfei Zhang, Xing Zhang, Jingwen Wang, Xing Jin, Miaomiao Xi, Jia Li

**Affiliations:** ^1^ Key Laboratory of Aerospace Medicine of Ministry of Education, School of Aerospace Medicine, Key Laboratory of Preventive Medicine of Ministry of Education, Fourth Military Medical University, Xi’an, Shaanxi 710032, China.; ^2^ Department of Pharmacy, Xijing Hospital, Fourth Military Medical University, Xi’an, Shaanxi 710032, China.; ^3^ Department of Cardiology, General Hospital of Central Theater Command, Wuhan, Hubei 430061, China.; ^4^ Senior Department of Ophthalmology, the Third Medical Center of PLA General Hospital, Beijing 100853, China.; ^5^ TANK Medicinal Biology Institute of Xi’an, Xi’an, Shaanxi 710032, China.; ^6^ Ministry of Education Key Lab of Hazard Assessment and Control in Special Operational Environment, Fourth Military Medical University, Xi’an, Shaanxi 712000, China.

## Abstract

It has been demonstrated that glutamine is a key player in boosting endothelial cell (EC) proliferation. However, despite its importance, the role of endothelial glutaminolysis in diabetes remains largely unexplored. Our research aimed to investigate the function of glutaminolysis in ECs within the context of diabetes and to evaluate the potential therapeutic effects of salvianolic acid B (SalB) and α-ketoglutarate (α-KG) on diabetic vascular complications. Histological analysis of skin wounds in diabetic patients revealed delayed restoration of vascularization and collagen synthesis during wound healing, accompanied by decreased glutaminase 1 (GLS1) expression and reduced colocalization with the EC marker platelet-endothelial cell adhesion molecule-1 (CD31). Additionally, a significant decline in GLS1 activity and expression was observed in ECs isolated from diabetic hearts. In vitro studies using cultured ECs demonstrated that exposure to high glucose and high fat (HGHF) reduced GLS1 expression and suppressed glutaminolysis, impairing EC proliferation and tube formation. These adverse effects were mitigated by treatment with SalB or supplementation with α-KG plus nonessential amino acids (NEAAs). Among diabetic mice subjected to myocardial ischemia/reperfusion (MI/R), SalB administration or α-KG supplementation promoted myocardial revascularization and improved cardiac dysfunction. Notably, endothelial-specific GLS1 deletion in mice blocked the beneficial effects afforded by SalB but not those afforded by α-KG. Furthermore, SalB administration accelerated angiogenesis and cutaneous wound healing in diabetic mice, and these influences were removed by pharmacological inhibition of GLS1 using bis-2-(5-phenylacetamido-1,3,4-thiadiazol-2-yl) ethyl sulfide (BPTES) or genetic deletion of endothelial GLS1. These findings indicate that defective endothelial glutaminolysis contributes to impaired angiogenesis and poor ischemic tissue repair in diabetes. Improving endothelial glutaminolysis by treatment with SalB or metabolic supplementation with α-KG promotes angiogenesis and ischemic tissue repair in diabetic mice, emphasizing the possibility of GLS1 as a treatment target.

## Introduction

Diabetes becomes a well-established hazard factor for cardiovascular disease and relates to vascular complications. These complications include impaired wound healing, nephropathy, neuropathy, retinopathy, and adverse outcomes related to myocardial ischemia/reperfusion (MI/R) [1]. Impaired ischemia-driven vasculogenesis has emerged as a hallmark of several diabetic vascular complications, such as suboptimal restoration and delayed wound healing following MI/R injury [2]. Angiogenesis is a dynamic and complex process that involves the mobilization and homing of endothelial cells (ECs) to sites of tissue damage [3,4]. This process significantly contributes to both re-endothelialization and neo-angiogenesis [5]. ECs respond to stimuli such as wounds by escaping dormancy, rapidly proliferating and invading hypoxic or ischemic tissues, forming novel vessels from established ones, and finally formulating a mature vascular network for the purpose of reestablishing vascular support [6]. Additionally, ECs serve as metabolic gatekeepers by adapting oxygen and nutrient delivery to meet the metabolic demands of tissues through angiogenesis [7,8]. They play a critical role in ensuring that tissues receive adequate supplies of oxygen and nutrients, which is crucial to sustain cell function and tissue health. In MI/R injury, ECs are particularly important [9]. Studies have shown that ECs possess distinct metabolic characteristics that are finely tuned to support their unique functions [10]. Fatty acid oxidation, glycolysis, and, more recently, glutamine/asparagine metabolism act as vital modulators of EC metabolism, significantly impacting angiogenesis in disease and health [11]. Thus, a deeper understanding of the mechanisms underlying angiogenesis could pave the way for innovative approaches to preventing and managing diabetic vascular diseases.

Glutamine is a crucial metabolic substrate that contributes to sustaining cellular bioenergetics, protein synthesis, and biosynthetic precursor generation [11,12]. Glutaminase 1 (GLS1) is the main isoform of glutaminase expressed in ECs, and the initial enzyme in the glutamine catabolism pathway, facilitating the conversion of glutamine to ammonia and glutamate. Subsequently, glutamate is metabolized into α-ketoglutarate (α-KG) by glutamate dehydrogenase (GLUD). This metabolic sequence is crucial for cellular bioenergetics and biosynthesis, particularly in ECs, where it plays a significant role in supporting angiogenesis and vascular health [13]. Glutamine metabolism plays a vital role in EC sprouting through tricarboxylic acid (TCA) cycle anaplerosis, redox homeostasis, macromolecule production, and endoplasmic stress control. Recent studies have highlighted that EC metabolic reprogramming is a key driver of angiogenesis to tissue ischemia, a process that is significantly dysregulated by diabetes [14,15]. A dual-blind, placebo-controlled trial has reported that glutamine addition beneficially affects glucose control, systolic blood pressure, and obesity in patients with diabetes [16]. Our group previously participated in a study that used genetic modification and ^13^C tracing methods to clarify glutamine metabolism in ECs. The results showed that glutamine provides most carbons in the TCA cycle of ECs and contributes to lipid biosynthesis via reductive carboxylation. EC-specific deletion in mice of GLS1 weakened the angiogenesis effect. In cell culture, glutamine deprivation or GLS1 inhibition significantly prevented EC proliferation [17]. Another article published around the same time further confirmed that depriving ECs of glutamine or inhibiting GLS1 leads to impaired angiogenesis and cell migration, and reduces pathological ocular angiogenesis. Inhibition of glutamine metabolism in ECs impairs TCA cycle anaplerosis, macromolecule production, and redox homeostasis [18]. However, it remains unclear whether endothelial glutamine metabolism is altered in diabetes and whether regulating endothelial glutamine metabolism can improve diabetes-induced angiogenesis impairments.

In recent years, there has been an increasing interest among researchers in exploring the potential of traditional Chinese herbs for promoting blood circulation and angiogenesis. Among them, *Salvia miltiorrhiza* Bge. (Danshen) has been recognized as a representative traditional Chinese medicine that enhances organ blood supply and is widely used in the treatment of cardiovascular and cerebrovascular diseases [19,20]. Salvianolic acid B (SalB) is one of the most abundant and active ingredients in the hydrophilic components of Danshen and has been shown to possess anti-apoptotic, anti-inflammatory, and vasodilatory effects after MI/R [21,22]. However, the precise mechanisms underlying SalB’s therapeutic effects against ischemic heart diseases in the diabetic state remain unclear. Herein, we report that defective endothelial glutamine metabolism is associated with diabetes-related angiogenic impairments post-MI/R and impaired wound healing. Moreover, we provide evidence that SalB administration improves endothelial glutamine metabolism, leading to enhanced angiogenesis in diabetic hearts and improved wound healing.

## Results

### GLS1 expression was decreased and reduced colocalization with the EC marker CD31 in skin wound healing of patients with diabetes after tissue injury

**Fig. 1.**
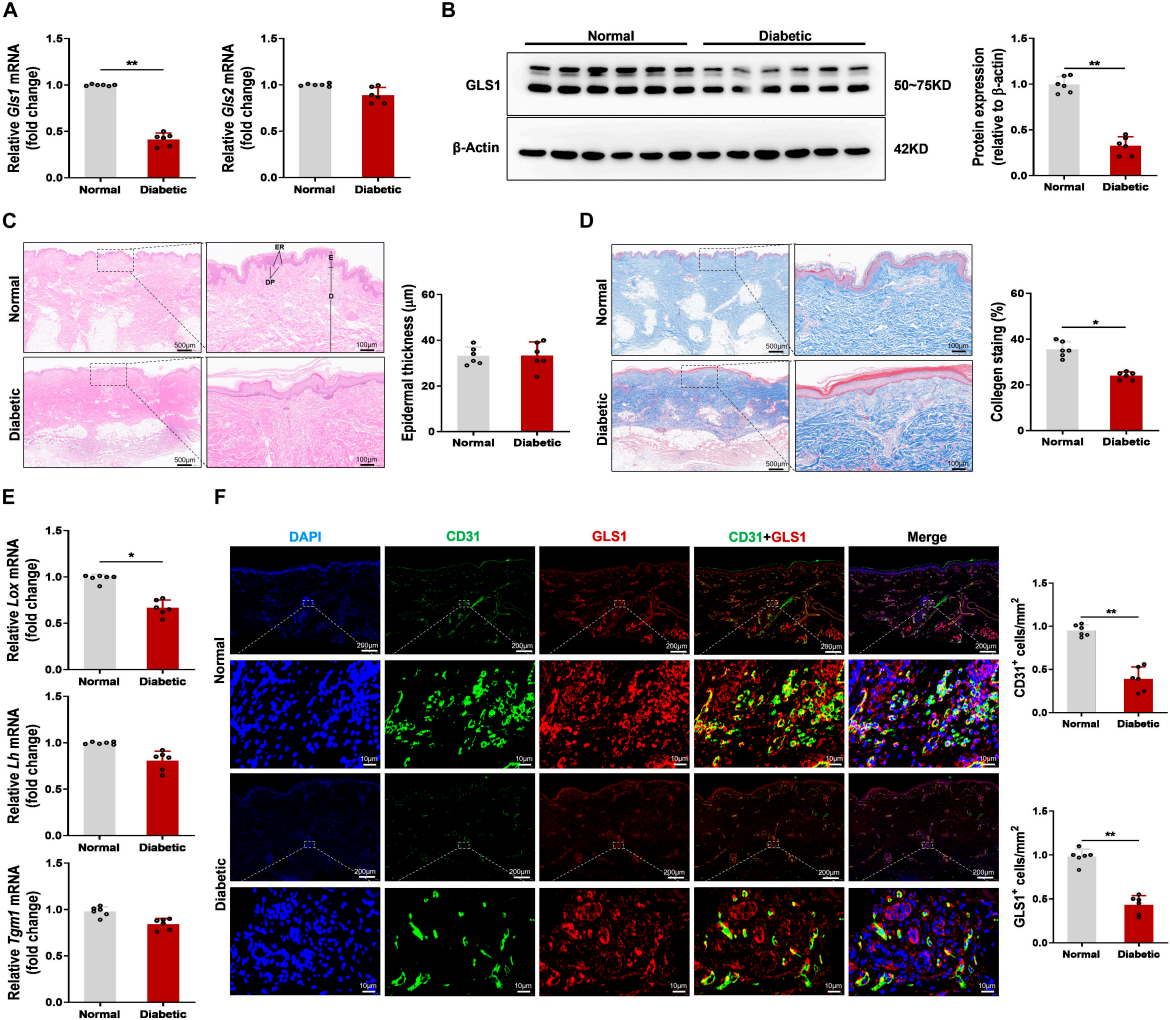
Glutaminase 1 (GLS1) expression was decreased and reduced colocalization with the endothelial cell (EC) marker platelet-endothelial cell adhesion molecule-1 (CD31) in skin wound healing of patients with diabetes after tissue injury. (A) Relative mRNA expression of *Gls1* and *Gls2* in human skin wound from diabetic and nondiabetic patients at day 3 after acute trauma. The data were standardized to the reference gene *18s rRNA*. (B) Representative blot images and quantitative analysis of GLS1 expression among diabetic and nondiabetic patients at day 3 after acute trauma. (C) Representative hematoxylin and eosin (H&E) images in human skin wound from diabetic and nondiabetic patients at day 3 after acute trauma. The full-thickness skin showed the epidermis (E) and the dermis (D). Arrows indicated the epidermal ridges (ERs) and the dermal papillae (DP). Scale bar, 500 μm (left) or 100 μm (right). (D) Representative Masson trichrome staining in human skin wound from diabetic and nondiabetic patients at day 3 after acute trauma. Dark blue staining indicates abundant collagen. Scale bar, 500 μm (left) or 100 μm (right). (E) Relative mRNA expression of *lysyl oxidases* (*Lox*), *lysyl hydroxylases* (*Lh*), and *transglutaminase 1* (*Tgm1*) in human skin wound from diabetic and nondiabetic patients at day 3 after acute trauma. The data were standardized to the reference gene *18s rRNA*. (F) Representative immunofluorescence images showed that GLS1 (red) colocalized with the EC marker CD31 (green) in human skin wound sections from diabetic and nondiabetic patients at day 3 after acute trauma. Scale bar, 200 μm (top) or 10 μm (bottom). *n* = 6/group, 6 diabetic patients and 6 matched nondiabetic patients were construed. Values are expressed as mean ± SEM. *P < 0.05; **P < 0.01.

Injured healing frequently triggers the progression of chronic wounds among diabetic patients, characterized by weak vascularization and intensified inflammatory reaction failing in suitably resolving. This condition highlights main defects during the processes participating in tissue remodeling and wound healing [23,24]. To obtain perceptions of the abnormality mechanisms in diabetic wound healing, we first collected human skin wound undergoing skin transplantation 3 d after acute trauma in patients with or without diabetes (patient information displayed in Table S1). We checked the level of *Gls* among nondiabetic and diabetic patients at day 3 after acute trauma of skin wound. Using quantitative real-time polymerase chain reaction (qRT-PCR) analysis, we found that *Gls1*, not *Gls2*, was decreased in the skin wound of diabetic patients (Fig. 1A). Likewise, immunoblots exhibited that the level of GLS1 protein was reduced within human skin wound from diabetic patients (Fig. 1B). Indeed, re-epithelialization and wound contraction become 2 of the dominant processes involved in wound healing of skins. As shown in Fig. 1C, after 3 d of acute trauma, hematoxylin and eosin (H&E) staining evaluation of nondiabetic patients did not show better re-epithelization characteristics compared with diabetic patients. However, in nondiabetic patients, all typical epidermal layers can be identified, consisting of the epidermis and the dermis, with the prominent epidermal ridges (ERs) and the dermal papillae (DPs) at their junction, along with the stratum corneum. On the contrary, in the epidermis of diabetic patients, it can be clearly observed that the nucleus is in a shrunken state (Fig. 1C). Masson trichrome staining revealed that the characteristic basket-weave orientation of collagen, typical of normal skin, was preserved in acute trauma skin samples collected 3 d post-injury from nondiabetic and diabetic patients, but compared with the skin wound of nondiabetic patients, collagen synthesis of wound in diabetic patients becomes slightly slower and less (Fig. 1D). We suspect that the less staining can also be due to deficient or impaired collagen cross-linking in diabetic patients after trauma; therefore, the tissue cannot produce more than that. In addition, in the absence of injury, the skin tissue of diabetic and nondiabetic patients did not change much (Fig. S1A and B). As shown in Fig. 1E, the mRNA levels of enzymes promoting collagen synthesis were analyzed. Three days post-acute trauma, collagen synthesis in nondiabetic patients was slightly higher compared to that in diabetic patients (Fig. 1E). To further scrutinize angiogenesis in diabetic patients, immunofluorescence staining analysis on platelet-endothelial cell adhesion molecule-1 (CD31) and α-smooth muscle actin (α-SMA) was implemented to rate the formation and maturation of blood vessels surrounding the wound regions. Actively stained vessels of α-SMA and CD31 among nondiabetic patient wounds were considerably higher than in diabetic patients (Fig. S2A and B). Briefly, the analysis uncovered abnormalities within skin wound healing among diabetic patients. Although no remarkable disparity occurred during the re-epithelialization process, the abnormalities were characterized by insufficient neovascularization and a postponed recovery of collagen synthesis.

Then, we employed immunofluorescence staining to check the GLS1 test among human skin wound sections from diabetic and nondiabetic patients. It was discovered that the positive expression of GLS1 among diabetic patients was remarkably lower than that in nondiabetic patients (Fig. S2C). Immunofluorescence further revealed that GLS1 was colocalized with CD31, a marker of ECs, and the expression level was significantly reduced in skin wound from diabetic patients (Fig. 1F).

### Glutaminolysis was impaired in diabetic ECs and restored by SalB treatment

**Fig. 2.**
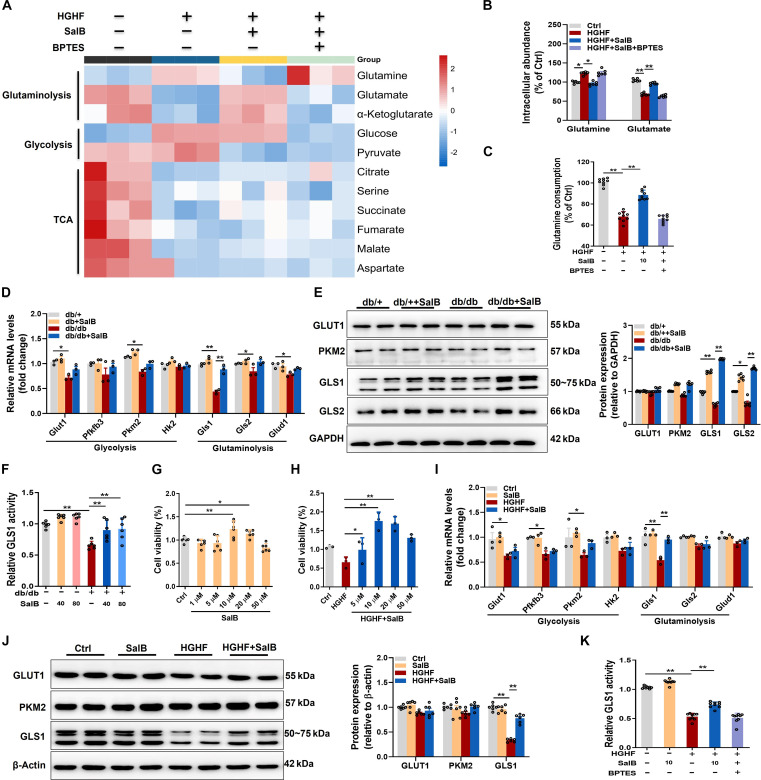
Glutaminolysis was impaired in diabetic ECs and restored by salvianolic acid B (SalB) treatment. (A to C and G to K) Human umbilical vein endothelial cells (HUVECs) were fixed in high glucose [25 mM, high glucose (HG)] and palmitic acid [400 μM, high fat (HF)] medium or control medium (5 mM glucose, 20 mM mannitol, Ctrl) treated with SalB (10 μM), with or without bis-2-(5-phenylacetamido-1,3,4-thiadiazol-2-yl) ethyl sulfide (BPTES; 5 μM) for 48 h. (A) Heatmap showing discrepant metabolites after HUVECs were exposed to HGHF medium or Ctrl mannitol, treated with SalB (10 μM), with or without BPTES (5 μM) for 48 h. Blue (red) denotes the comparative down (up) expressions of glutaminolysis, glycolysis, and tricarboxylic acid (TCA) cycle intermediates compared with cells treated with the Ctrl. *n* = 3. (B) Intracellular glutamine and glutamate among HUVECs. (C) Glutamine consumption in the cultured medium. (D) Relative mRNA level of indicated genes engaging in glycolysis [*glucose transporter type 1* (*Glut1*), *phosphofructokinase-2/fructose-2,6-bisphosphatase-3* (*Pfkfb3*), *pyruvate kinase isozyme type M2* (*Pkm2*), and *hexokinase 2* (*Hk2*)] and glutaminolysis [*Gls1*, *Gls2*, and *glutamate dehydrogenase 1* (*Glud1*)] in cardiac microvascular ECs isolated from db/db or db/+ mice treated with or without SalB (40 and 80 mg/kg/d) for 2 weeks. Data were standardized to the reference gene *18s rRNA*. (E) Representative blot images and quantitative analysis on indicated protein (GLUT1, PKM2, GLS1, GLS2) expression. (F) GLS1 activity in cardiac microvascular ECs isolated from db/db or db/+ mice. (G) CCK-8 assay of SalB on HUVECs in 48 h. (H) CCK-8 assay of SalB on HUVECs treated with HGHF within 48 h. (I) Relative mRNA level of indicated genes engaging in glycolysis (*Glut1*, *Pfkfb3*, *Pkm2*, and *Hk2*) and glutaminolysis (*Gls1*, *Gls2*, and *Glud1*) in HUVECs. Data were standardized to the reference gene *18s rRNA*. (J) Representative blot images and quantitative analysis indicated protein (GLUT1, PKM2, GLS1) level. (K) GLS1 activity in HUVECs. Values are expressed as mean ± SEM. *P < 0.05; **P < 0.01.

To investigate potential metabolic alterations in ECs in diabetes, we measured a panel of candidate metabolites reflecting the activities of glutaminolysis, glycolysis, and the TCA circulation across different treatment groups. Metabolite set enrichment analysis of differentially abundant metabolites showed that high-glucose and high-fat (HGHF) treatment induced significant alterations in glutaminolysis, characterized by a marked rise in glutamine expressions and a reduction in glutamate expressions. It is suggested that inhibited glutaminolysis under diabetic conditions with an abnormal enrichment of glutamine content. Interestingly, SalB, a highly biologically active compound found in the traditional Chinese medicine *S. miltiorrhiza* Bge, treatment can significantly promote the catabolism of glutamine to glutamate and improve the deficiency of endothelial glutaminolysis in diabetic state (Fig. 2A). It is worth noting that there are also differences in the effects on other intracellular metabolites, such as glucose and pyruvate levels in glycolysis, demonstrating that in addition to the inhibition of glutaminolysis, the normal glucose metabolism pathway may also be disrupted, and the underlying mechanism needs to be further explored (Fig. S3). To further confirm the pattern of glutamine catabolism and glutamine consumption in cell culture media, the changes of intracellular glutamine and glutamate contents were particularly measured on human umbilical vein endothelial cells (HUVECs) treated or untreated with HGHF. We discovered that intracellular glutamine significantly increased, but glutamate decreased, in HUVECs exposed to HGHF for 48 h. In line with the metabolomic results, SalB treatment partially down-regulated intracellular glutamine levels, together with a rise in glutamate levels, exerting a more pronounced effect on glutaminolysis in HUVECs exposed to HGHF, but blocked by cotreatment with the GLS1-specific inhibitor BPTES (Fig. 2B). A trait of cells experiencing glutamine-dependent anaplerosis becomes a raised rate of glutamine consumption [25]. By directly measuring glutamine consumption in the culture medium, the results showed that exposure to HGHF reduced glutamine consumption in HUVECs by approximately 32% to 35%. These effects were partially restored by SalB treatment and also blocked by cotreatment with BPTES (Fig. 2C). On the whole, data suggest that impaired glutaminolysis occurred among diabetic individuals.

To further investigate the specific changes in ECs glycolysis and glutaminolysis under diabetic conditions, we isolated microvascular ECs from the hearts of db/db diabetic mice and their control db/+ mice. Among the ECs of db/db mice, mRNA levels of glycolysis-related genes, including *Glut1*, glycolytic regulator phosphofructokinase-2/fructose-2,6-bisphosphatase-3 (*Pfkfb3*), and *Pkm2*, except for the rate-limiting enzyme hexokinase 2 (*Hk2*), were significantly reduced. Additionally, the enzyme GLS1/GLS2 serves as an essential modulator of the first and rate-limiting step of glutaminolysis among mitochondria [26] and catabolizes glutamine into glutamate. Indeed, it was discovered that the mRNA level of *Gls1*, a critical enzyme of glutaminolysis, decreased by approximately 50%, and the mRNA level of *Gls2* and glutamate dehydrogenase 1 (*Glud1*), a vital modulator of glutaminolysis, also decreased significantly compared with those in ECs of db/+ mice. More interestingly, administration with SalB for 2 weeks significantly increased *Gls1* mRNA expression in ECs of db/db mice, but the impact on glycolytic-related genes was less pronounced (Fig. 2D). Meanwhile, Western blot analysis revealed that the protein level of GLS1 and GLS2 among ECs also rose significantly after SalB treatment, with an increase in microvascular EC of db/db mice relative to that of db/+ mice (Fig. 2E). Given that GLS1 is the gatekeeper for the conversion of glutamine to glutamate, to determine whether SalB affected GLS1 activity, we continued to examine the GLS1 activity of microvascular ECs in the hearts of different treatment groups. EC activity among db/db mice was prominently decreased compared to ECs among db/db mice, and as expected, GLS1 activity was partially restored when different doses of SalB treatment were administered (Fig. 2F).

In vitro, a cell counting kit-8 (CCK-8) test was implemented to measure the maximal nontoxic concentration of SalB on HUVECs treated or untreated with HGHF within 48 h. As shown in Fig. 2G and H, the concentration of SalB in our study was chosen as 10 or 20 μM. Consistent with in vivo data, HGHF exposure for 48 h significantly reduced *Gls1* mRNA (Fig. 2I) and protein expression (Fig. 2J) and inhibited GLS1 activity (Fig. 2K) in cultured HUVECs, all of which were rescued by SalB treatment. These results suggest that GLS1 expression and activity are impaired in diabetic ECs and that SalB has the potential to promote GLS1 expression and activity. Furthermore, these findings indicate that glutaminolysis is inhibited in diabetic conditions, which is largely improved by SalB treatment.

### SalB modulates GLS1-mediated glutamine metabolism remodeling in diabetic ECs by targeting

**Fig. 3.**
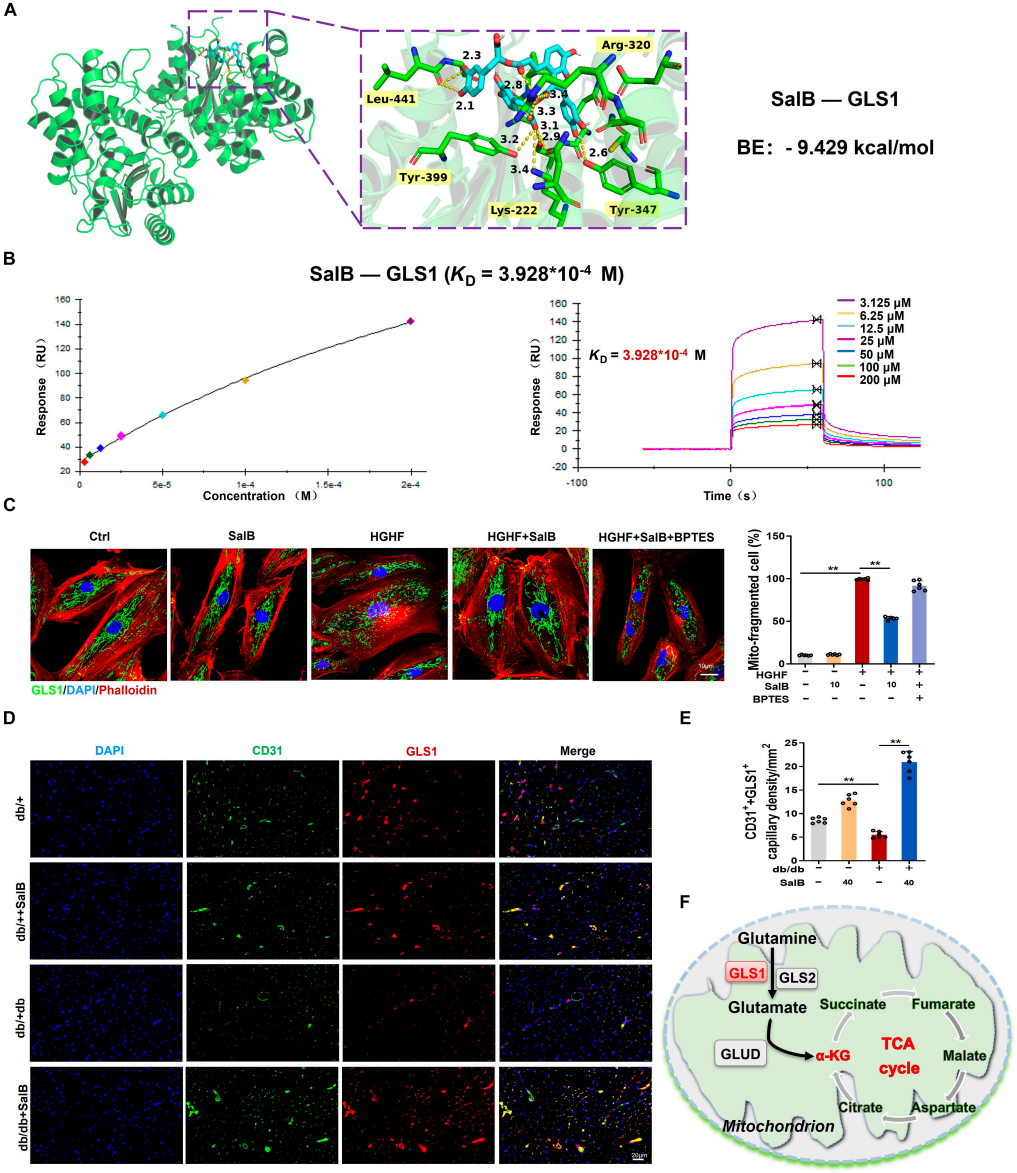
SalB modulates GLS1-mediated glutamine metabolism remodeling in diabetic ECs by targeting. (A) Molecular docking analysis of SalB binding to GLS1 with binding energies of −9.429 kcal/mol. (B) The interaction between SalB and GLS1 was determined by SPR assay. (C) Immunofluorescence staining of GLS1 (green), phalloidin (red), and DAPI (blue) in HUVECs. Scale bar, 10 μm. (D and E) Representative immunofluorescence images showed that GLS1 (red) colocalized with the EC marker CD31 (green) in hearts segregated from db/db or db/+ mice processed with or without SalB (40 mg/kg/d) for 2 weeks. Scale bar, 20 μm. (F) Schematic representation of mitochondrial glutamine–GLS1–glutamate metabolic process. *n* = 6/group. Values are denoted as mean ± SEM. **P < 0.01.

Previous studies conducted by the research group have confirmed that glutaminolysis, started by GLS1-mediated conversion of glutamine into glutamate, is associated with disorders in EC angiogenesis [17]. To further determine whether SalB can regulate glutamine metabolism of ECs in diabetic state by targeting GLS1, we performed molecular docking analysis between SalB and the putative targets GLS1 and GLS2. As demonstrated by 3D structure display, SalB was predicted to form hydrogen bonds at Arg^320^, Leu^441^, Tyr^399^, Lys^222^, and Tyr^347^ of GLS1, with a binding energy of −9.429 kcal/mol, and at Gly^404^, Ser^343^, Lys^354^, Gly^348^, and Asn^351^ of GLS2, with a binding energy of −7.332 kcal/mol. These results indicate a strong interaction between SalB and GLS1 proteins compared to GLS2 (Fig. 3A and Fig. S4). Furthermore, to further confirm the binding between SalB and its key effective target GLS1, we conducted a surface plasmon resonance (SPR) experiment. Experimentally, the GLS1 protein was immobilized on the chip, and 7 concentration gradients of SalB solution were flowed over the chip to assess the binding response using SPR detection. As shown in Fig. 3B, SalB exhibited concentration-dependent binding to the GLS1 protein, and the GLS1 protein binds SalB with a dissociation constant (*K*_D_) of 3.928 × 10^−4^ M.

GLS is expressed within the mitochondria. In our preliminary experiment, we stained the mitochondria, cytoskeleton, and nucleus using MitoTracker Red (red), GLS (green), phalloidin (red), and 4′,6-diamidino-2-phenylindole (DAPI) (blue), separately. Figure 3C and Fig. S5 show that there is a cluster of mitochondria among cells, with the nucleus positioned centrally among them, and the cytoskeleton surrounding this mitochondrial cluster. The cell boundaries are clearly visible and distinct, even in the absence of specific nuclear or cytoplasmic markers. Using MitoTracker Red alone makes it straightforward to distinguish one cell from its neighbors. Typically, a cluster of mitochondria highlighted by the stain corresponds to a single cell. In addition, we can also clearly see that GLS1 are located in the mitochondria of ECs, and their morphology is mainly manifested as elongated tubules in a highly interconnected network. Then we further determined the targeting relationship between SalB and GLS1. HUVECs were cultured with or without SalB in the Ctrl medium for 48 h, and mitochondrial morphology typically presents as elongated tubules forming a highly interconnected network. However, following 48-h stimulation with HGHF, mitochondria exhibited fragmentation, and the expression of GLS1 was significantly diminished, while both of which were blocked by SalB treatment (Fig. 3C). Moreover, immunofluorescence costaining with CD31, a marker of ECs, revealed diminished fluorescent intensity for both GLS1 and CD31 among hearts of db/db mice, a diabetic model, and SalB treatment can promote the coexpression of GLS1 and CD31 in diabetic mice (Fig. 3D and E). Given that GLS1 serves as the gatekeeper for glutamine changing into glutamate—a critical step for glutamine entering the TCA circulation (Fig. 3F), these findings suggest that SalB can improve glutamine metabolism remodeling in diabetic ECs by targeting GLS1 regulation.

### SalB treatment or α-KG plus NEAA supplementation rescued EC proliferation and migration in HGHF condition

**Fig. 4.**
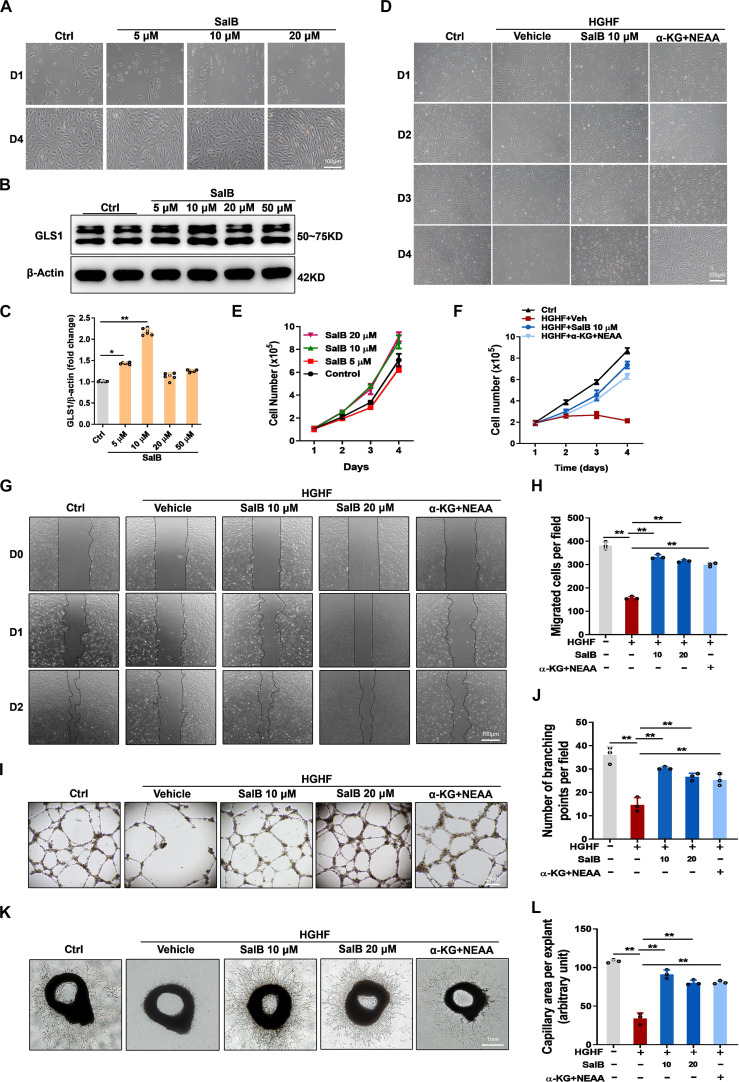
SalB treatment or α-ketoglutarate (α-KG) plus nonessential amino acids (NEAAs) supplementation rescued EC proliferation and migration in HGHF condition. HUVECs were exposed to HGHF medium or Ctrl medium treated with vehicle (Veh), SalB (5, 10, and 20 μM) or cell-permeable α-KG (2 mM) plus NEAAs (0.2 mM). (A and E) Phase contrast images and growth curves of HUVECs following 4-d culture within Ctrl medium with the increasing concentrations of SalB (5, 10, and 20 μM). Scale bar, 100 μm. (B and C) Representative blot images and quantitative analysis of GLS1. (D and F) Phase contrast images (original magnification, ×40) and growth curves of HUVECs during 4 d of different treatments. Scale bar, 100 μm. (G and H) Representative images (original magnification, ×40) of cell scratch test and quantitative analysis of HUVECs during 48 h of different treatments. Scale bar, 100 μm. (I and J) Tube-forming capacity of HUVECs after 48 h of different treatments. Representative images (original magnification, ×10) describing the forming of capillary-like tube constructions by HUVECs on Matrigel. Each tube was photographed, and the number of branching was quantified by using ImageJ. Scale bar, 25 μm. (K and L) Capillary sprouting from mouse aortic explants with different treatments for 4 d. Each explant was photographed, and the area of capillary outgrowth was quantified by using ImageJ. Scale bar, 1 mm. Values are represented as mean ± SEM from 3 independent experiments. *P < 0.05; **P < 0.01.

EC proliferation and migration are hallmark of endothelial function [27] and are blunted in diabetic [28]. We first observed that SalB enhanced HUVEC growth in a concentration-dependent manner, as evidenced by cell counting assays (Fig. 4A and E). Additionally, 10 μM SalB was able to significantly promote GLS1 expression, strongly justifying the concentration selection in this study (Fig. 4B and C). Next, to explore the influence of SalB on EC development and transfer in the context of diabetes, we subjected HUVECs to HGHF substrate with or without SalB. Furthermore, it is shown that glutamine metabolism regulates the yield of adenosine triphosphate (ATP) via the intermediate product α-KG that enters the TCA circulation as an anaplerotic carbon origin and an important point in the anaplerotic response [29]. Given the inhibition of glutaminolysis under HGHF conditions, we further tested whether supplementation with α-KG plus NEAAs could rescue EC proliferation and migration [30,31]. Indeed, α-KG plus NEAA supplementation restored HGHF-induced inhibition of EC proliferation and migration. Interestingly, SalB treatment further promoted EC proliferation and migration in HGHF conditions (Fig. 4D and F to H). Additionally, we assessed tube-forming ability in HUVECs cultured on matrigel, which was significantly impaired by HGHF exposure. However, SalB treatment or α-KG plus NEAA supplementation significantly enhanced tube-forming ability in ECs exposed to HGHF (Fig. 4I and J). Consistent with these findings, aortic explants from mice exposed to HGHF exhibited markedly impaired capillary sprouting, which was largely rescued by SalB treatment or α-KG plus NEAA supplementation (Fig. 4K and L). Collectively, our data suggest that SalB and α-KG plus NEAA treatment effectively rescue EC proliferation and migration under diabetic conditions.

### SalB administration or α-KG supplementation improved cardiac dysfunction in diabetic mice post-MI/R

**Fig. 5.**
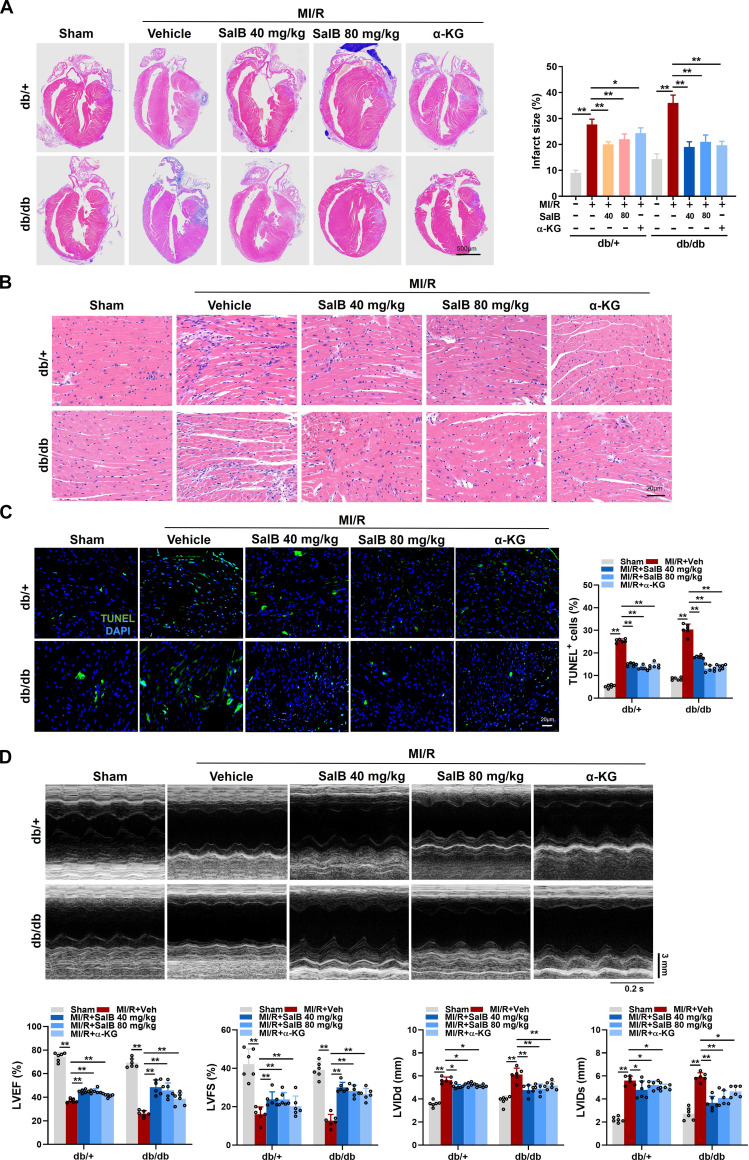
SalB administration or α-KG supplementation improved cardiac dysfunction in diabetic mice post-myocardial ischemia/reperfusion (MI/R). (A) Heart cross-sections in db/+ and db/db mice processed with Veh, SalB (40 and 80 mg/kg/d), or α-KG (10 mg/kg/d) at day 7 post-MI/R. Quantitative analysis is displayed on the right. Scale bars, 500 μm. (B) Representative H&E images in the myocardial infarct border zone of db/+ and db/db mice with different treatments at day 7 post-MI/R. Scale bar, 20 μm. (C) Representative immunofluorescence images of terminal deoxynucleotidyl transferase–mediated deoxyuridine triphosphate nick end labeling (TUNEL) staining and quantitative analysis within the myocardial infarct border zone of db/db and db/+ mice with different treatments at day 7 post-MI/R. Scale bars, 20 μm. (D) Representative echocardiographic images and quantified left ventricular ejection fraction (LVEF), left ventricular fractional shortening (LVFS), left ventricular internal diameter in diastole (LVIDd), and left ventricular internal diameter in systole (LVIDs) of db/db mice with different treatments at day 7 post-MI/R. *n* = 6/group. Values are denoted as mean ± SEM. *P < 0.05; **P < 0.01.

SalB, one of the richest bioactive polyphenolic components in *S. miltiorrhiza* Bge, demonstrates therapeutic and defensive influences against a variety of illnesses [32]. In addition, evidence has shown that α-KG supplementation reduced the incidence rate, resulting in health benefits among mice [33]. Hence, the influence of SalB administration or α-KG supplementation was examined in diabetic mice post-MI/R. Indeed, we observed that db/db mice exhibited more significant myocardial disorganization and more inflammatory cell infiltration, and the infarct size was raised at day 7 post-MI/R relative to db/+ mice. SalB administration and α-KG supplementation improved the cardiac morphology and structure prominently among db/db mice relative to db/+ mice (Fig. 5A and B). As illustrated in Fig. 5C, in contrast to the control hearts of db/+ mice 7 d post-MI/R, the apoptotic indicator was significantly elevated among diabetic hearts of db/db mice at the same time point, and administration of SalB and supplementation of α-KG significantly reduced apoptotic index in diabetic hearts on day 7 post-MI/R, with a decrease of apoptotic index in db/db mice relative to that of db/+ mice on day 7 post-MI/R. Moreover, SalB administration or α-KG supplementation significantly ameliorated cardiac dysfunction among db/db mice on day 7 post-MI/R, as exhibited by improved left ventricular ejection fraction (LVEF) and left ventricular fractional shortening (LVFS) and decreased left ventricle at end-diastole (LVIDd) and left ventricle at end-systole (LVIDs), relative to vehicle treatment or db/+ mice (Fig. 5D). Findings indicate that adverse pathological changes in db/db mice post-MI/R were effectively mitigated by SalB administration or α-KG supplementation, compared to those observed in db/+ mice post-MI/R.

### Administration of SalB rescued EC proliferation and promoted angiogenesis in diabetic mice post-MI/R by targeting GLS1

**Fig. 6.**
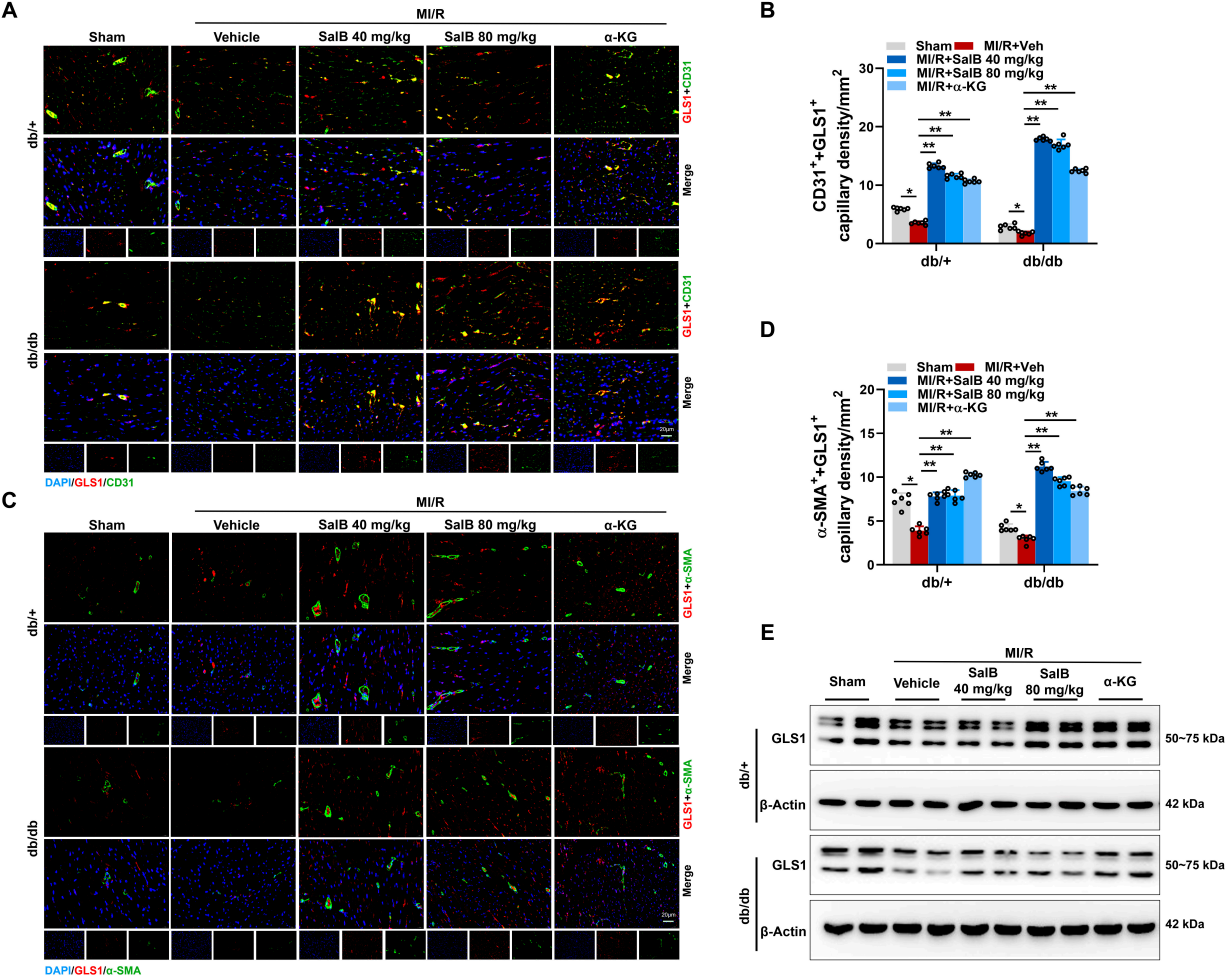
Administration of SalB rescued EC proliferation and promoted angiogenesis in diabetic mice post-MI/R by targeting GLS1. (A and B) Representative immunofluorescence images showed that GLS1 (red) colocalized with the EC marker CD31 (green), and quantitative analysis in the myocardial infarct border zone of db/db and db/+ mice treated with Veh, SalB (40 and 80 mg/kg/d), or α-KG (10 mg/kg/d) at day 7 post-MI/R. Scale bar, 20 μm. (C and D) Representative immunofluorescence images showed that GLS1 (red) colocalized with the vascular smooth muscle cell marker α-smooth muscle actin (α-SMA; green) and quantitative analysis in the myocardial infarct border zone of db/db and db/+ mice with different treatments at day 7 post-MI/R. Scale bars, 20 μm. *n* = 6/group. (E) Representative blot images of GLS1. Values are denoted as mean ± SEM. *P < 0.05; **P < 0.01.

Because neovascularization is essential for the repair of heart damage, we aimed to determine whether angiogenesis was adversely impacted in diabetic mice following MI/R. Additionally, we also investigated the impact of administration of SalB or supplementation of α-KG on angiogenesis. Indeed, we found more CD31-positive cells and α-SMA-positive cells within the infarct border area of db/db mouse heart administration of SalB or supplementation of α-KG on day 7 post-MI/R relative to those treated with vehicle or db/+ mice (Fig. S6A and B), suggesting that neovascularization may occur in the infarct border zone upon both SalB administration and α-KG supplementation. Findings deeply indicate that SalB or α-KG could be used as novel pro-angiogenic factors to facilitate cardiac repair in diabetic conditions. To further dissect that glutamine metabolism induces EC proliferation and promotes neovascularization after MI/R in diabetic, we costained GLS1, the first enzyme of glutamine catabolism, with EC marker CD31 and small vessel marker α-SMA by immunofluorescence. Findings exhibit that, relative to db/+ mice, the coexpression of GLS1 and CD31 and the coexpression of GLS1 and α-SMA were remarkably down-regulated among db/db mice at 7 d after MI/R. SalB administration prominently promoted the coexpression of both, and the coexpression of MI/R among diabetic mice exceeded that among nondiabetic mice (Fig. 6A to D). Furthermore, the protein expression of GLS1 was measured. Relative to the control hearts of db/+ mice, GLS1 level was remarkably reduced among diabetic hearts of db/db mice. SalB administration could significantly promote the protein expression of GLS1 after MI/R in db/db mice (Fig. 6E). Collectively, these findings suggest that glutaminolysis is defective in diabetes, which contributes to impaired angiogenesis and myocardial dysfunction post-MI/R. SalB administration or α-KG supplementation can rescue EC proliferation and boost angiogenesis in diabetic mice post-MI/R by targeting GLS1.

### Reconstitution of GLS1 and its co-administration of SalB further rescued angiogenesis in diabetic mice post-MI/R

**Fig. 7.**
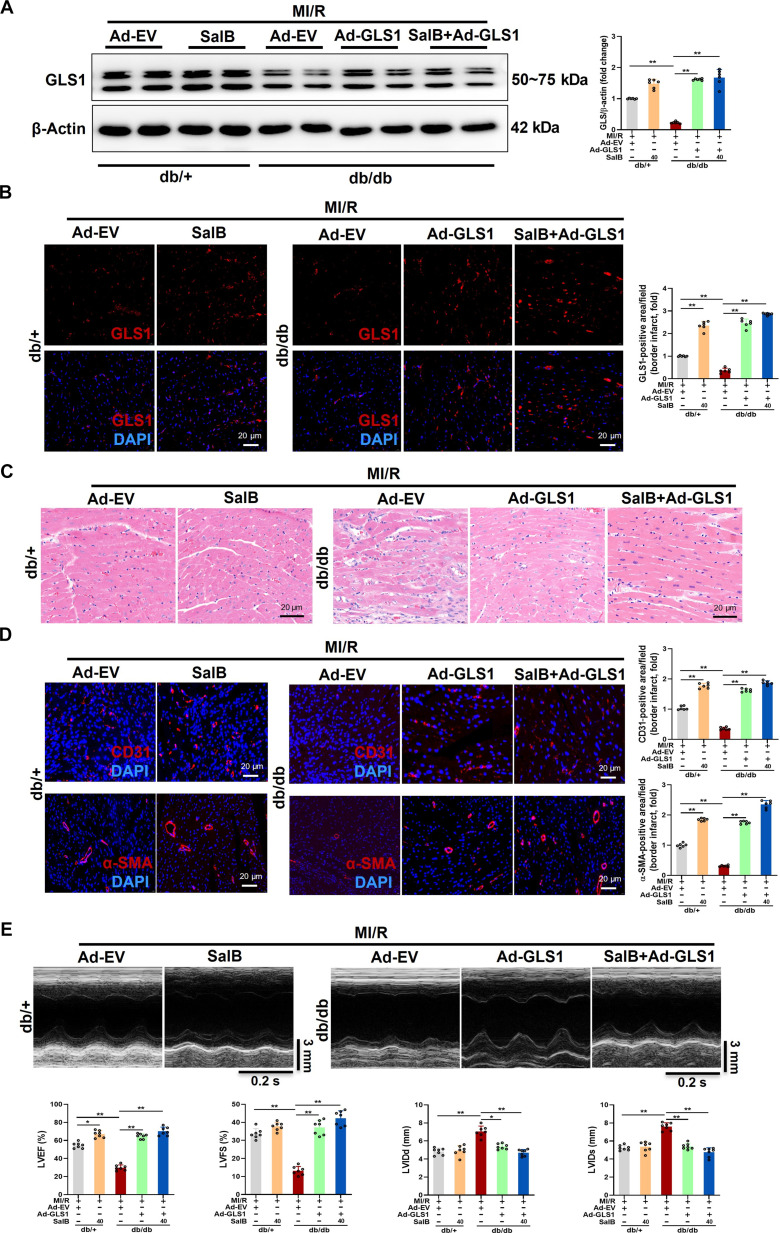
Reconstitution of GLS1 and its co-administration of SalB further rescued angiogenesis in diabetic mice post-MI/R. (A) Representative blot images and quantitative analysis of GLS1 expression. (B) Representative immunofluorescence images of GLS1 in the myocardial infarct border zone of db/+ and db/db mice treated with Ad-EV (control adenovirus), Ad-GLS1 (recombinant adenovirus encoding GLS1), SalB (40 mg/kg/d), or SalB + Ad-GLS1 at day 7 post-MI/R. Quantitative analysis is displayed on the right. Scale bar, 20 μm. (C) Representative H&E images in the myocardial infarct border zone of db/+ and db/db mice with different treatments at day 7 post-MI/R. Scale bar, 20 μm. (D) Representative immunofluorescence images of the EC marker CD31 and the vascular smooth muscle cell marker α-SMA in the myocardial infarct border zone of db/+ and db/db mice with different treatments at day 7 post-MI/R. Quantitative analysis is displayed on the right. *n* = 6/group. Scale bar, 20 μm. (E) Representative echocardiographic images and quantified LVEF, LVFS, LVIDd, and LVIDs of db/+ and db/db mice with different treatments at day 7 post-MI/R. *n* = 7/group. Values are denoted as mean ± SEM. *P < 0.05; **P < 0.01.

To further determine whether the reconstitution of GLS1 exerts beneficial effects on angiogenesis in diabetic mice post-MI/R, adenoviral vectors encoding GLS1 were injected intramyocardially into db/db mice during MI/R modeling and administration of SalB ​at the same time. As shown in Fig. 7A, myocardial GLS1 expression was increased approximately 7-fold among db/db mouse hearts on day 7 post-MI/R after GLS1-expressing adenovirus (Ad-GLS1) injection relative to empty vector-expressing adenovirus (Ad-EV) injection. The administration of SalB at the same time was about 9-fold exceeding that of Ad-EV. GLS1 immunofluorescence staining further confirmed the robust and widespread increase in GLS1 expression throughout nearly the entire left ventricular area at day 7 post-MI/R following injection. This suggests that intramyocardial adenovirus injection successfully achieved transmural expression of GLS1 in the mouse hearts. Moreover, SalB administration promoted the significant expression of GLS1 (Fig. 7B). Histopathological results also demonstrated that both Ad-GLS1 injection and its co-administration with SalB significantly improved myocardial pathological damage (Fig. 7C). Immunofluorescence staining further confirmed more CD31-positive cells and α-SMA-positive cells within the infarct border region of mouse hearts with Ad-GLS1 injection at day 7 post-MI/R relative to those of Ad-EV injection. Similarly, co-administration with SalB significantly increased EC number and enhanced blood vessel density. SalB administration also significantly increased the number of ECs and vessel density among hearts of db/+ mice at day 7 post-MI/R (Fig. 7D). Moreover, echocardiography indicated that reconstitution of GLS1 improved cardiac function among db/db mice on day 7 post-MI/R as indicated by raised LVEF and LVFS and reduced LVIDd and LVIDs. There is no doubt that co-administration with SalB further significantly promoted cardiac repair (Fig. 7E). Collectively, our data indicate that on the basis of intramyocardial injection of Ad-GLS1, SalB treatment further induced neovascularization and facilitated cardiac repair.

### Endothelial-specific GLS1 deletion blocked angiogenic effects in mice post-MI/R afforded by SalB but not by α-KG

**Fig. 8.**
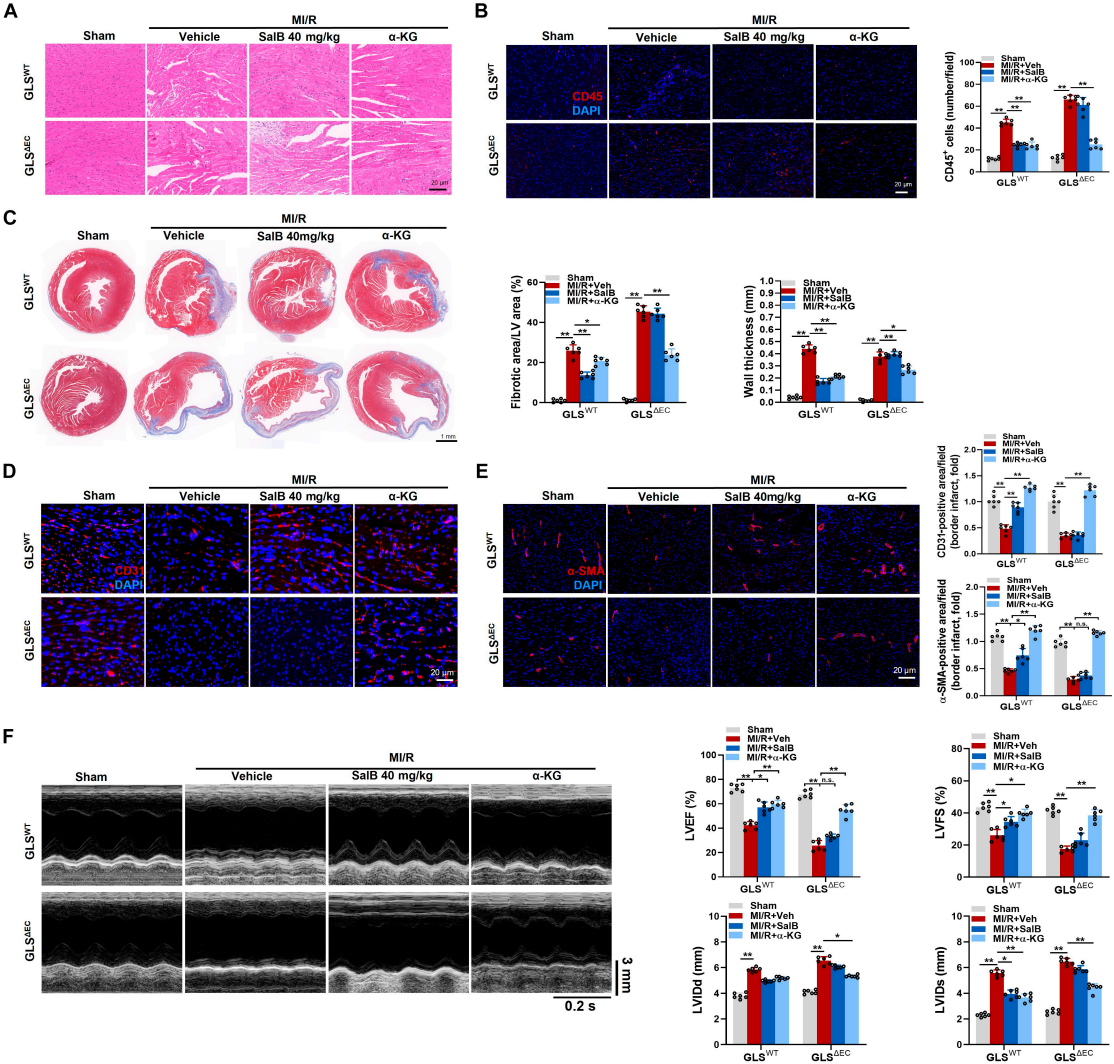
Endothelial-specific GLS1 deletion blocked angiogenic effects in mice post-MI/R afforded by SalB but not by α-KG. (A) Representative H&E images in the myocardial infarct border zone of GLS^ΔEC^ and GLS^WT^ mice treated with Veh, SalB (40 mg/kg/d), or α-KG (10 mg/kg/d) at day 7 post-MI/R. Scale bar, 20 μm. (B) Representative immunofluorescence images of CD45 and quantitative analysis in the myocardial infarct border zone of GLS^ΔEC^ and GLS^WT^ mice with different treatments at day 7 post-MI/R. Scale bar, 20 μm. (C) Representative Masson trichrome staining of cardiac tissue and quantitative analysis of the fibrotic area and the wall thickness of GLS^ΔEC^ and GLS^WT^ mice with different treatments at day 7 post-MI/R. Scale bar, 1 mm. (D) Representative immunofluorescence images of the EC marker CD31 and quantitative analysis in the myocardial infarct border zone of GLS^ΔEC^ and GLS^WT^ mice with different treatments at day 7 post-MI/R. Scale bar, 20 μm. (E) Representative immunofluorescence images of the vascular smooth muscle cell marker α-SMA and quantitative analysis in the myocardial infarct border zone of GLS^ΔEC^ and GLS^WT^ mice with different treatments at day 7 post-MI/R. Scale bars, 20 μm. (F) Representative echocardiographic images and quantified LVEF, LVFS, LVIDd, and LVIDs of GLS^ΔEC^ and GLS^WT^ mice with different treatments at day 7 post-MI/R. *n* = 6/group. Values are represented as mean ± SEM. *P < 0.05; **P < 0.01.

To explore the function of glutaminolysis in the angiogenic influences of SalB, GLS^ΔEC^ and GLS^WT^ mice were sensitive to MI/R treatment with SalB. The histopathological examination and CD45^+^ immunofluorescence staining of heart tissues exhibited that the hearts from the sham-operated groups of both GLS^WT^ and GLS^ΔEC^ mice exhibited a clear structure, with minimal inflammatory cell infiltration and negligible pathological changes. However, at day 7 post-MI/R, it became evident that the GLS^ΔEC^ mice displayed an obscured tissue architecture and disorganized myocardial fibers, along with an essential rise in inflammatory cell infiltration relative to the GLS^WT^ mice. SalB administration largely preserved cardiac morphology and structure in GLS^WT^ mice but not in GLS^ΔEC^ mice, while α-KG supplementation showed beneficial effects in both groups (Fig. 8A and B). Masson trichrome staining revealed that no remarkable difference occurred in the gross morphology of sham-operated hearts between GLS^ΔEC^ and GLS^WT^ mice. By contrast, SalB administration significantly reduced cardiac fibrosis areas and wall thickness at the infarct area of GLS^WT^ mice at 7 d following MI/R. Interestingly, the protective effects were still completely inhibited in mice with endothelial-specific GLS1 deletion. Conversely, α-KG supplementation reduced cardiac fibrosis areas and wall thickness at the infarct area in both GLS^WT^ and GLS^ΔEC^ mice (Fig. 8C). Similarly, at day 7 post-MI/R, SalB administration significantly increased CD31-positive cells and α-SMA-positive cells within the myocardial infarct border area of GLS^WT^ mice, indicating enhanced EC number and blood vessel density. However, these effects were completely abolished in mice with endothelial-specific GLS1 deletion. Conversely, α-KG supplementation increased CD31-positive cells and α-SMA-positive cells among GLS^WT^ and GLS^ΔEC^ mice (Fig. 8D and E). Cardiac function was also assessed by echocardiography, revealing that GLS^ΔEC^ mice exhibited exacerbated cardiac dysfunction compared to GLS^WT^ mice at day 7 post-MI/R. SalB administration significantly improved cardiac function in GLS^WT^ mice but not in GLS^ΔEC^ mice, while α-KG supplementation showed beneficial effects in both groups (Fig. 8F). These results suggest that endothelial GLS1-driven glutaminolysis is a key player in cardiac angiogenesis post-MI/R, and that improved endothelial glutaminolysis contributes greatly to the angiogenic and cardioprotective effects of SalB.

### Beneficial effects afforded by SalB on wound healing of diabetic mice were abolished by BPTES

**Fig. 9.**
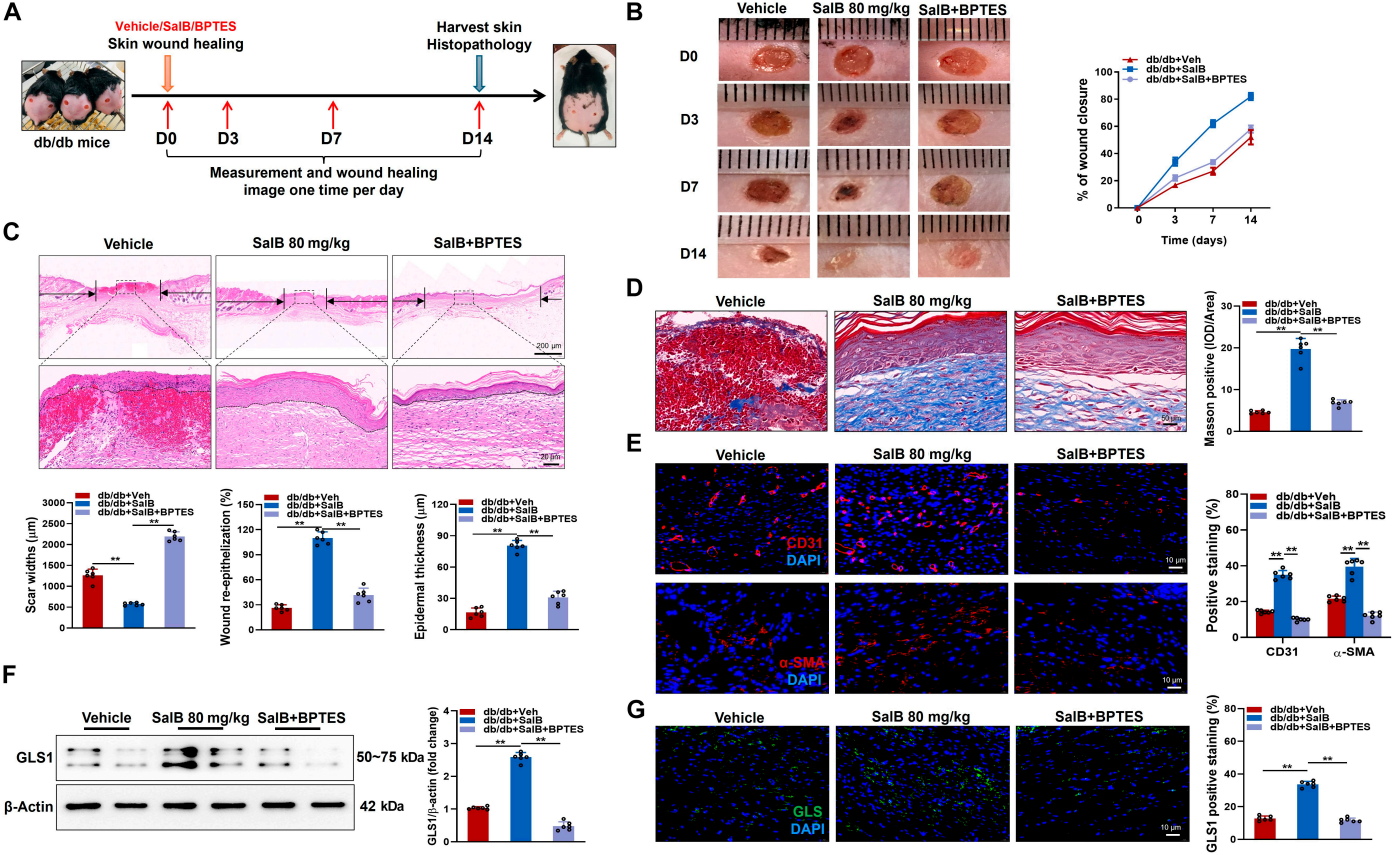
Beneficial effects afforded by SalB on wound healing of diabetic mice were abolished by BPTES. (A) Timeline of wound treatment process. Initiation of treatment with Veh, SalB (80 mg/kg/d), or BPTES (5 mg/kg/d) was considered to be day 0, with wounds photographed and measured each day until day 14. (B) Representative images and quantification of wound closure of db/db mice with different therapies at days 0, 3, 7, and 14 following cutaneous punch biopsy. Veh, SalB, or BPTES was subcutaneously injected surrounding the wounds at 4 injection positions. (C) Representative H&E images and quantitative analysis on the scar widths, the degree of re-epithelization, and the epidermal thickness of db/db mice with different treatments at day 14 after cutaneous punch biopsy. The black arrows denote the scar edges, and dotted lines stand for the boundary between epidermis and dermis. Scale bar, 200 μm (top) or 20 μm (bottom). (D) Representative Masson trichrome staining in wound sections of db/db mice with different treatments at day 14. Scale bar, 50 μm. (E) Representative immunofluorescence images of the EC biomarker CD31 (red) and the vascular smooth muscle cell marker α-SMA (red) among wound sections of db/db mice with different treatments at day 14. Quantitative analysis is shown on the right. Scale bar, 10 μm. (F) Representative blot images and quantitative analysis of GLS1. (G) Representative immunofluorescence images of the GLS1 (green) in wound sections of db/db mice with different treatments at day 14. Quantitative analysis is shown on the right. Scale bar, 10 μm. *n* = 6/group. Values are denoted as mean ± SEM. **P < 0.01.

To examine the influences of SalB on angiogenesis among tissues outside of the heart, the full-thickness cutaneous wounds occurred on backs of db/db mice. These wounds were then treated with subcutaneous injections of SalB, either alone or in combination with BPTES. The progress of wound closure was monitored by measuring wound size on days 0, 3, 7, and 14 (Fig. 9A). Findings indicated that relative to the vehicle-treated group, SalB treatment significantly accelerated wound healing, with complete healing observed at approximately 14 d after injury. However, this effect was abolished by cotreatment with the GLS1-specific inhibitor BPTES (Fig. 9B). The histopathological analysis revealed that, relative to the vehicle-treated group, the SalB experimental group exhibited not only an accelerated healing rate but also a thicker epidermis in the skin sections of the treated mice (Fig. 9C). Furthermore, on postoperative day 14, Masson trichrome staining (which highlights collagen in blue) revealed a greater density of collagen fibers in the SalB treatment group relative to the vehicle-treated group. As to the vehicle-treated group, the skin surrounding the wound exhibited disorganized collagen architecture and reduced collagen levels. The collagen of SalB-treated mice was remarkably up-regulated; however, the beneficial changes were also completely abolished by cotreatment with BPTES (Fig. 9D). Similarly, immunofluorescence staining of α-SMA and CD31 was implemented to calculate the density of novel blood vessels at wound sites among db/db mice. Findings denoted that SalB therapy increased the quantity of CD31 and α-SMA actively stained cells, indicating the promotion of angiogenesis (Fig. 9E). In addition, immunoblots showed that SalB treatment also increased GLS1 protein expression (Fig. 9F). However, this beneficial effect of SalB was abrogated by cotreatment with BPTES (Fig. 9F). Similarly, this finding was further corroborated by immunofluorescence staining of GLS1 (Fig. 9G). Collectively, these findings indicate that SalB enhances angiogenesis and wound healing among diabetic mice, with the effect being predominantly mediated by the activation of GLS1.

### Endothelial deletion of GLS1 abolished the beneficial effects of SalB but not α-KG on cutaneous wound healing in mice

**Fig. 10.**
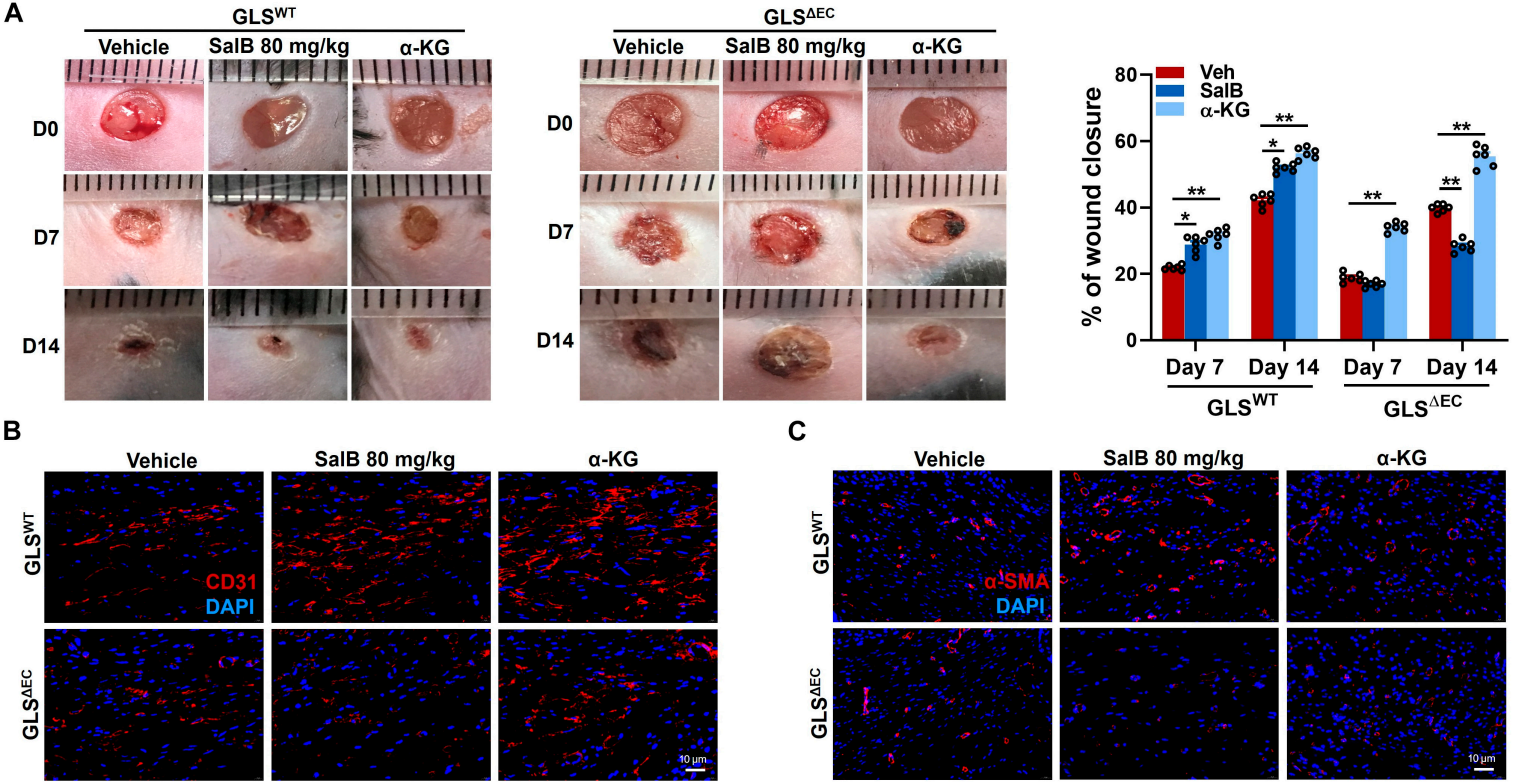
Endothelial deletion of GLS1 abolished the beneficial effects of SalB but not α-KG on cutaneous wound healing in mice. (A) Representative images and quantification of wound closure of GLS^ΔEC^ and GLS^WT^ mice with different therapies at days 0, 7, and 14 following cutaneous punch biopsy. SalB (80 mg/kg/d) or α-KG (10 mg/kg/d) was subcutaneously injected around the wounds at 4 injection sites. (B and C) Representative immunofluorescence images of the EC marker CD31 (red) and the vascular smooth muscle cell marker α-SMA (red) among wound sections of GLS^ΔEC^ and GLS^WT^ mice with different treatments at day 14. Scale bar, 10 μm. *n* = 6/group. Values are denoted as mean ± SEM. *P < 0.05; **P < 0.01.

To further elucidate the crucial role of GLS1 in wound healing properties of SalB, the full-thickness cutaneous wounds were induced on backs of GLS^WT^ and GLS^ΔEC^ mice, before subcutaneous injections of SalB or α-KG. The influence of these therapies on wound closure was rated by measuring wound region on days 0, 7, and 14 post-wounding. As depicted in Fig. 10A, SalB administration and α-KG supplementation both accelerated wound closure in GLS^WT^ mice, as evidenced by reduced wound regions at day 7 and 14 post-wounding compared to vehicle-treated mice. Conversely, in GLS^ΔEC^ mice, only α-KG supplementation facilitated wound closure, whereas SalB administration failed to exhibit any significant effects. Similarly, immunofluorescence staining of α-SMA and CD31 was implemented to calculate the density of novel blood vessels at wound sites of GLS^WT^ and GLS^ΔEC^ mice. Findings manifested that SalB therapy raised the quantity of CD31 and α-SMA positively stained cells in GLS^WT^ mice, indicating the promotion of angiogenesis. In mice that specifically knocked out GLS1 in ECs, the angiogenesis-promoting effect of SalB was also inhibited compared to α-KG (Fig. 10B and C). These observations suggest that endothelial-specific deletion of GLS1 completely abrogates the wound healing potential of SalB but not α-KG in mice.

## Discussion

In this study, an analysis of wound healing in human skin demonstrated that diabetic patients experience delayed recovery of collagen synthesis and angiogenesis. Additionally, it was further observed that the expression of GLS1, a key enzyme in glutamine metabolism, was significantly reduced in the skin wounds of diabetic patients, accompanied by decreased colocalization with CD31, an EC marker. Interestingly, continuing to examine metabolic differences in ECs between diabetic and nondiabetic states, we found defects in the angiogenesis, glutaminolysis, and wound healing signaling pathways, all of which are mediated by dysregulation of GLS1 signaling. We functionally validated these diabetes-related deficiencies using both in vitro cell culture and in vivo mouse models of angiogenesis, including a vessel tube formation assay in cell culture, a mouse model of MI/R, and a model of skin wound healing. Our results demonstrated reduced expression and activity of GLS1 in microvascular ECs of diabetic mice and in cultured HUVECs exposed to HGHF. Importantly, treatment with SalB was found to up-regulate the expression and activity of GLS1, promoting glutaminolysis under diabetic conditions. This enhancement in glutaminolysis facilitated ischemia-driven angiogenesis, improved cardiac function post-MI/R, and accelerated wound healing in diabetic mice. Molecular docking experiments and SPR technology confirmed that SalB mediates angiogenesis in diabetes by targeting GLS1 activation. In the absence of endothelial-specific GLS1 or upon treatment with the GLS1-specific inhibitor BPTES, SalB-induced angiogenesis was significantly inhibited. However, these beneficial effects were not influenced by α-KG supplementation. These findings underscore the potential of SalB administration to regulate glutamine metabolism through targeted GLS1 modulation, presenting a promising strategy for treating diabetic microvascular complications. On the one hand, α-KG supplementation is attributed to its role as a precursor of glutamate in glutamine metabolism, driving the synthesis of glutamine; on the other hand, α-KG supplementation can form a dynamic cycle with glutamate and glutamine to maintain the intracellular carbon–nitrogen balance. Thus, when GLS1 was specifically inhibited, the above beneficial effects of SalB administration were inhibited, while α-KG was not.

Glutamine, a plentiful and versatile amino acid, is a basic player in cardiovascular health and disease. Research has shown that it can help mitigate some hazard risk factors regarding cardiovascular disease, like hyperlipidemia, hypertension, diabetes, obesity, and glucose intolerance [34]. Interestingly, patients with diabetes exhibit altered glutamine metabolism, characterized by reduced serum concentrations of glutamine and α-KG. Preclinical research has indicated that targeting glutaminolysis may hold promise as a therapeutic approach for managing diabetic complications, highlighting the need for further in-depth investigation in this area. Presently, research has underscored the role of endothelial glutamine metabolism as an emerging and crucial modulator of vascular progression and etiological angiogenesis [11]. In particular, 2 studies have explored how glutamine metabolism impacts EC proliferation, migration, and sprouting, underscoring its importance in maintaining EC homeostasis and promoting angiogenesis [17,18]. SalB, a natural antioxidant extracted from the roots and rhizomes of Danshen, exhibits a broad spectrum of biological activities. It holds significant promise in the treatment of cardiovascular diseases, including atherosclerosis and MI/R [35]. Additionally, research indicates that SalB may be beneficial for the management of various other conditions, because of its potent antioxidant properties [32]. Our previous studies have confirmed that SalB promotes macrophage polarization by inhibiting mammalian target of rapamycin complex 1 (mTORC1)-dependent glycolysis, thereby ameliorating inflammatory responses and cardiac dysfunction following MI/R [36]. In 2022, a comprehensive study further highlighted the therapeutic potential of SalB in mitigating diabetes-induced mitochondrial and endothelial dysfunction by down-regulating mitophagy and apoptosis in ECs [22]. Liu et al. [37] study demonstrated that SalB improves vascular endothelial dysfunction by influencing the bone morphogenetic protein 4-reactive oxygen species cycle in diabetic mice, playing a crucial role in the treatment of diabetic vasculopathy. Furthermore, in a study focusing on diabetic cardiomyopathy, the administration of SalB was found to increase the levels of vascular endothelial growth factor receptor 2 and vascular endothelial growth factor A in a dose-dependent manner by inhibiting insulin-like growth factor binding protein 3. This inhibition promoted angiogenesis both in vivo and in vitro, consequently reducing myocardial fibrosis and remodeling [21]. Consequently, a progressive amount of evidence indicates that SalB can become an ideal candidate compound for treating diabetic vascular complications by improving endothelial dysfunction and promoting angiogenesis. In this study, based on an earlier article that our research group participated in and published, which deeply clarified the metabolic characteristics of glutamine in ECs [17], we further explored the impact of glutamine metabolism on the occurrence and development of diabetes [38]. Our first constructive finding was that on the 3 d after acute trauma, compared with nondiabetic patients, the expression and protein levels of GLS1, the initial enzyme, key enzyme, and rate-limiting enzyme of glutamine metabolism, in the skin of diabetic patients were decreased, and the colocalization with CD31, a marker of ECs, was significantly reduced. This suggests that glutamine metabolism in ECs is abnormal under diabetic conditions, which may affect wound healing and tissue remodeling to a certain extent. Further exploration of the potential metabolic regulation changes of ECs in diabetes revealed that quantitative analysis of various metabolites involved in intracellular glutamine metabolism, glycolysis, and TCA cycle across different treatment groups showed that when HUVECs were exposed to HGHF conditions, intracellular glutamine levels increased, while glutamate levels significantly decreased, which seems to be ascribed to the suppression of GLS1 expression and viability by HGHF. Similar results were also observed in cardiac microvascular ECs isolated from diabetic mice (db/db), which is associated with injured angiogenesis in diabetes. Interestingly, SalB treatment could promote the decomposition of glutamine into glutamate by targeting and mechanistically enhancing the expression and activity of GLS1, thereby improving the abnormal glutamine metabolism in ECs under diabetic conditions. In addition, α-KG, as a crucial product of glutamine metabolism, functions as an intermediate fuel in the TCA cycle. It plays a pivotal role in various biological processes, including antioxidant defense [39], energy production (without the need for complex preliminary metabolic steps) [40], and epigenetic modification [41]. Furthermore, it is demonstrated that α-KG mitigates hyperlipidemia-induced dyslipidemia [42] and endothelial destruction [43]. On this basis, our in vitro experiments demonstrated that both SalB administration and α-KG plus NEAA supplementation rescued HGHF-induced impairments in various cellular processes including proliferation, migration, and capillary sprouting in ECs. Different models in vivo confirmed that SalB administration or α-KG supplementation accelerated angiogenesis and cutaneous wound healing among diabetic mice, which eliminated the beneficial effects provided by SalB rather than α-KG by pharmacological inhibition of GLS1 or genetic deletion of endothelial GLS1. Various in vivo models, including the mouse MI/R model and wound healing experiments, further confirmed that SalB administration or α-KG supplementation can improve ischemic angiogenesis and cardiac dysfunction after MI/R in diabetic mice, as well as promote skin wound healing. Particularly intriguing was our observation that when we performed endothelial-specific deletion of GLS1 or pharmacologically inhibited GLS1 expression, the beneficial effects of SalB were negated, whereas those of α-KG remained unaffected. Additionally, reconstituting GLS1 and combining it with SalB restored angiogenesis in diabetic mice post-MI/R. These results sufficiently demonstrate that endothelial GLS1-driven glutaminolysis plays a crucial role in cardiac angiogenesis post-MI/R and wound healing. The administration of SalB targets the activation of GLS1, thereby regulating glutaminolysis in ECs, which may help realize its therapeutic potential for diabetic microvascular complications. Furthermore, exogenous α-KG supplementation can directly participate in the TCA cycle, modulate glutamine metabolism, enhance energy utilization efficiency, rescue HGHF-induced impairments in proliferation and migration of HUVECs, as well as promote angiogenesis in diabetic states.

In summary, our study has reported on the effects of SalB on glutaminolysis in ECs under conditions of diabetes-induced injury. These data have indicated that SalB improves ischemic angiogenesis post-MI/R and accelerates wound healing in diabetic mice via regulating glutamine metabolism by boosting the level and activity of GLS1 among ECs of diabetes. These findings provide compelling new evidence for SalB as a potential therapeutic agent for preventing and treating various chronic complications in diabetic patients, such as cardiovascular complications, diabetic foot, and refractory wound healing, which provide an important reference for the clinical conversion and treatment upgrade of SalB. Furthermore, targeting glutaminolysis by regulating GLS1 may be an underlying treatment tactic for diabetic angiogenesis and ischemic tissue repair.

To sum up, this paper employed various models to further elucidate the protective effects of SalB on the vascular complications associated with diabetes by targeting GLS1 and regulating glutamine metabolism. This work also enriches our understanding of the pharmacological mechanisms through which SalB addresses clinical complications. Moreover, our research group also conducted a comprehensive analysis and in-depth evaluation of the current research on SalB using scientometrics in 2024 [32]. SalB has been shown to be safe and effective, with minimal side effects. Its modified preparation, *S. miltiorrhiza* polyphenolic acid salt for injection (comprising 80% SalB), is extensively used in clinical settings. However, due to SalB’s high molecular weight, its absolute oral bioavailability is low, significantly limiting its therapeutic potential. Therefore, developing SalB combinations, novel dosage forms, innovative vectors, and alternative delivery methods represents both a challenge and an opportunity for us and other researchers. Previous studies have demonstrated that the combined use of SalB and ginsenoside Rg1 can produce a synergistic effect, enhancing therapeutic efficacy while reducing toxic reactions. This combination demonstrates excellent pharmacological benefits in the treatment of heart diseases [44,45]. Additionally, innovative dosage forms and new carriers (e.g., nanoparticles, hydrogel carriers, and freeze-dried powders) improve the stability and bioavailability of SalB, enabling it to act directly on therapeutic targets, independent of other physical factors. It also avoids first-pass liver effects and hepatorenal toxicity, as well as reduces gastrointestinal reactions [46–48]. New delivery methods for SalB include in vivo implantation, intramuscular injection, intramuscular disc injection, and direct application to damaged skin or disease sites. These innovative approaches pave the way for developing high-efficacy, long-acting SalB formulations, offering promising therapeutic options for various diseases. However, there remains a significant gap in clinical research regarding SalB combination therapy, new dosage forms, and novel drug delivery methods, and how to translate these basic theoretical studies into clinical practice is a difficult problem to be solved. In the future, we can leverage emerging technologies such as artificial intelligence (AI), molecular modeling, and drug-target docking, alongside advanced biological methods like genomics, metabolomics, and cell sequencing. These tools will enable us to further explore and precisely determine the positioning and transformational potential of SalB in the clinical treatment of various diseases. In addition to the limitations of this study, we must also acknowledge that there is a significant gap between building animal models and human disease development, as well as the challenges in translating findings from animal studies to clinical applications. On the one hand, animal models often induce diseases rapidly through artificial interventions (e.g., drugs, diet modifications, or surgical procedures), whereas human diseases typically develop over time due to a complex interplay of environmental, genetic, and lifestyle factors. For instance, in this study, db/db mice (featuring a leptin receptor spontaneous mutation) were utilized to simulate a diabetes model. However, human type 2 diabetes represents a gradual metabolic disorder process, leading to essential differences in both pathological mechanisms and processes between the 2. On the other hand, there are notable distinctions between animals and humans concerning gene expression, metabolic pathways, immune systems, and more. For example, atherosclerosis does not naturally occur in mice, and although we created a MI/R model using ligation of the left anterior descending artery (LAD), replicating the intricate pathology of the disease remains challenging. Despite these limitations, animal models remain indispensable tools in medical research. In future studies, we plan to integrate various species or models according to experimental design to encompass a broader range of biological characteristics. By combining in vitro experiments (such as organoid cultures), computational models (like AI-driven predictions), and human cohort studies, we aim to elucidate new therapeutic targets for clinical diseases from multiple perspectives. This multidimensional approach promises to provide deeper insights into disease mechanisms and potential treatments.

## Conclusion

To summarize, our findings demonstrate that GLS1 inhibition and impaired glutaminolysis are contributing factors to the reduced ischemia-driven angiogenesis observed in diabetes. This implies that GLS1 might act as an underlying therapeutic target for enhancing angiogenesis in diabetic patients. Furthermore, our data reveal novel angiogenic effects of SalB, offering additional evidence for its potential utility in treating ischemic heart diseases and promoting diabetic wound healing.

## Materials and Methods

### Human sample collection

The procurement of human skin samples received approvals from the Medical Ethics Committee of Xijing Hospital, Fourth Military Medical University (no. KY20233600-1), and followed the principles formulated in the Declaration of Helsinki. Written consent was derived from the whole participants before sample gathering. Patients with untreated vascular diseases or invasive infections, including active cellulitis, abscesses, or osteomyelitis, characterized by systemic manifestations, were excluded from the study. For epidermal profiling, skin wound tissues were collected as surplus material from debridement procedures performed on patients undergoing skin transplantation 3 d post-acute trauma; these specimens would have been discarded otherwise. The cohort comprised nondiabetic individuals (4 men and 2 women, average age 49 years) and diabetic patients (5 males and 1 female, mean age 52 years). All clinical diagnoses pertained to lower extremity injuries caused by car accidents, which occurred prior to specimen collection at the Department of Burn and Dermatology, Xijing Hospital, Fourth Military Medical University. It is important to note that no experimental induction of trauma/injury was performed after specimen collection. Upon acquisition, wound samples were washed using phosphate-buffered saline (PBS) under aseptic conditions and subsequently bifurcated for discrepant analyses: One part was frozen swiftly in liquid nitrogen and preserved at −80 °C to analyze protein, while the other was immobilized in 4% paraformaldehyde for histological sectioning. Additional particulars regarding patient samples can be found in Table S1.

### Skin wound histological and immunofluorescence analysis

For histological assessments, 4-μm-thick sections from paraffin-embedded skin samples were formulated and sensitive to Masson trichrome staining as well as H&E staining by Servicebio (China). Microscopic images were captured through an Olympus BX71 microscope (Japan), with Image-Pro Plus 6 software (Media Cybernetics, Bethesda, USA) utilized for measuring the epidermis thickness and assessing the completeness of the newly formed skin among H&E-stained sections. The Masson trichrome staining highlighted collagen maturity with blue-stained collagen fibers.

To investigate the expression of GLS1, α-SMA, CD31, and immunofluorescence staining was performed on rehydrated paraffin sections. Slides were firstly vulnerable to antigen repair via microwave heating for 8 min. Following this, they were inhibited using 4% bovine serum albumin (BSA) for 30 min at 37 °C to minimize nonspecific binding. Primary antibodies against CD31 (ab28364, Abcam, diluted 1:800), α-SMA (ab5694, Abcam, diluted 1:800), and GLS1 (ab156876, Abcam, diluted 1:800) were utilized all night at 4 °C. Following washing through PBS, the slides were incubated using a horseradish peroxidase (HRP)-conjugated goat anti-rabbit secondary antibody (ab97075, Abcam, diluted 1:200) for 1 h at 37 °C. Staining was visualized using diaminobenzidine tetrahydrochloride and investigated through an optical microscope. The levels of CD31, α-SMA, and GLS1 were calculated by determining the percentage of positive-stained region to the whole tissue region through Image-Pro Plus 6 software [23,49,50].

### Animals

The experiments involving animals were conducted in compliance with the National Institutes of Health Guidelines for the Use of Laboratory Animals and were approved by the Committee on the Care and Use of Laboratory Animals of the Fourth Military Medical University. The mice were housed in individual ventilated cages at a constant temperature of 24 ± 2 °C and 45 ± 15% humidity, with a 12-h/12-h light–dark cycle (lights on 07:00 h, lights off 19:00 h), and were provided with ad libitum access to standard laboratory food and water [51].

Leptin receptor-deficient (db/db) mice at the age of 8 weeks old and their age-matched male littermate controls (db/+) originated in Changzhou Cavens Laboratory Animal Co. Ltd., located in Jiangsu, China. Mice harboring floxed alleles of the GLS1 gene (Gls*^lox/lox^*) along with transgenic mice expressing tamoxifen-inducible Cre recombinase under the VE-cadherin promoter (both strains on a C57BL/6J background) were supplied by Shanghai Model Organisms Center Inc., based in Shanghai, China. For the purpose of developing EC-specific GLS knockout (GLS^ΔEC^) mice and their corresponding wild-type controls (GLS^WT^), we crossed Gls*^lox/lox^* mice with the aforementioned transgenic mice showing tamoxifen-inducible Cre recombinase driven by the VE-cadherin promoter [17]. Tamoxifen (40 mg/kg) was offered to mice for 5 consecutive days, and the following experiments were performed 4 weeks later.

### Isolation of ECs

Db/db mice at the age of 12 weeks old and their control littermates were segregated stochastically into 6 groups, including db/+ group, db/db group, db/+ + SalB 40 mg/kg/d group, db/+ + SalB 80 mg/kg/d group, db/db + SalB 40 mg/kg/d group, and db/db + SalB 80 mg/kg/d group, with and without SalB treatment (by intraperitoneal injection) for 2 weeks; each group consisted of 6 mice (*n* = 6). Before the experiment ended (the mice were 14 weeks old), the cardiac microvascular ECs were isolated and gathered. Blinding was used in subsequent analyses.

 The night before, the Dynabead + anti-CD31 antibody solution was prepared. Each mouse required 2 μl of Dynabead, with 0.12 μl of anti-CD31 antibody on each Dynabead. After preparation, it was stored at 4 °C overnight. Mice were anesthetized using isoflurane, and their whole body was soaked in ethanol for disinfection. A total of 15 ml of precooled PBS was then perfused through the left ventricle. The right atrium was subsequently cut open to serve as an outlet for the PBS. After perfusion, the heart was excised in its entirety, chopped into smaller pieces, and rinsed with precooled PBS. Subsequently, the chopped cardiac tissue was quickly transferred into a sterile hood. A digestion mixture consisting of 5 mg/ml collagenase I (#LS004194, Worthington, Ohio, USA) and 700 μl of DN-25 (a deoxyribonuclease) in 7 ml of PBS was then added to the cardiac tissue. The digested cardiac tissue was incubated in a shake incubator at 250 rpm for 20 min. Following incubation, the mixture was filtered through a 100-μm filter. Cardiac tissue fragments retained on the filter were gently disrupted using the plunger of a 1-ml syringe. The filtrate was then resuspended in 10 ml of Dulbecco’s modified Eagle’s medium (DMEM, #11966025, Thermo Fisher Scientific, USA) supplemented with 10% fetal bovine serum (FBS, #F8318, Sigma-Aldrich), and then the above steps were repeated again. The filtrate was subsequently passed through a 40-μm filter, then resuspended in 10 ml of DMEM containing glutamine and 10% FBS. The suspension was then subjected to centrifugation at 200 rpm for 5 min at 4 °C. Following centrifugation, the supernatant was discarded. The reaction was stopped with 10% FBS within DMEM before passing through cell strainers to remove debris. Cell pellet was resuspended in DMEM (with Glutamax and 10% FBS) and plated within a 10-cm dish containing 0.1% gelatin in DMEM. After 45 min, the supernatant was collected, with cells resuspended in Dynabead + anti-CD31 antibody through 0.1% BSA (#10711454001, Sigma-Aldrich), brooded for 15 min on ice with gentle tapping, mounted on mag-bar, and washed with 0.1% BSA before resuspending in endothelial cell growth medium (EGM; #CC-3121 and #CC-4133, Lonza) using gelatin-coated for further experimentation.

### Culture of HUVECs

HUVECs within passages 3 to 5 were procured from Lonza and used in the study. Such cells were cultivated within eosin–methylene blue agar medium (EBM) added into EGM supplements and 10% FBS. To meet the experimental requirements, cell-permeable dimethyl-α-ketoglutarate (#349631, Sigma-Aldrich) and nonessential amino acids (NEAAs; 100×, #11140050, Thermo Fisher Scientific) were added at the specified concentrations. NEAAs were added to stimulate HUVEC growth and prolong the viability of EC culture. For the ease of presentation, dimethyl-α-ketoglutarate is further abbreviated as α-KG. In addition, SalB was initially dissolved in dimethyl sulfoxide (DMSO) and subsequently diluted in DMEM to prepare solutions for cell treatment. HUVECs were exposed to varying concentrations of SalB, and the incubation medium was changed every other day. 

### RNA extraction and qRT-PCR analysis

RNAs were obtained from cells or tissues through TRIzol reagent (Invitrogen, USA). In terms of mRNA detection, 1 μg of RNAs was reverse-transcribed into cDNA through the RevertAid First-Strand cDNA Synthesis Kit (Thermo Fisher Scientific, USA). Following this, qRT-PCR was implemented with SYBR Premix Ex Taq II (#RR820L, Takara Bio Inc., Shiga, Japan) on an ABI Prism 7900HT Sequence Detection System (Thermo Fisher Scientific, USA). Gene expressions were quantified in contrast with *18s rRNA* as a reference gene, figured out through the 2^−ΔΔCT^ method, and represented as fold change. Table S2 displays the primer sequences.

### Immunoblot analysis

Protein extraction was carried out using radioimmunoprecipitation assay lysis buffer, which contains 149 mM NaCl, 50 mM Tris (pH 7.5), 0.1% sodium dodecyl sulfate, 1% Nonidet P-40 substitute, 0.5% deoxycholic acid, and sodium salt, along with Roche protease and phosphatase inhibitors. Following separation by sodium dodecyl sulfate–polyacrylamide gel electrophoresis in decreased conditions, lysates were delivered to a polyvinylidene difluoride membrane and subjected to immunoblotting. Immunoblot analysis was conducted using primary antibodies against glucose transporter type 1 (GLUT1; ab14683, Abcam, Cambridge, UK), pyruvate kinase isozyme type M2 (PKM2; ab85555, Abcam), GLS1 (ab156876, Abcam), and β-actin (#4970S, Cell Signaling Technology, Danvers, MA) at dilutions of 1:1,000 and 1:2,000. Protein expression was visualized through Western ECL Substrate (Thermo Fisher Scientific). Each experiment was independently conducted 3 times.

### GLS1 enzyme activity measurement

The activity of the GLS1 enzyme was evaluated using a Glutaminase Assay Kit (#E-133, Biomedical Research Service Center, NY, USA), following the manufacturer protocol. Specifically, cells were integrated into a glutamine solution, followed by a 2-h incubation at 37 °C. Subsequently, the assay buffer was integrated into the samples, which underwent extra hour incubation at the same temperature. Optical density (OD) was then measured at 492 nm through one microplate spectrophotometer (Thermo Fisher Scientific). Each sample was made in triplicate, with enzyme activity quantified following the manufacturer’s guidelines. To confirm the reproducibility and robustness of the results, all assays were repeated independently at least thrice.

### Glutamine and glutamate level measurements

HUVECs were subjected to elevated glucose [25 mM, high glucose (HG)] and palmitic acid [400 μM, high fat (HF)] levels [high-glucose and high-fat (HGHF) medium] or control medium (5 mM glucose, 20 mM mannitol, Ctrl medium) for 48 h, with concurrent treatment of 10 μM SalB [36] or 5 μM GLS1-specific inhibitor bis-2-(5-phenylacetamido-1,3,4-thiadiazol-2-yl) ethyl sulfide (BPTES) [52] (#SML0601, Sigma-Aldrich). Notably, to be more clear about the effects of SalB, SalB was not pre-incubated with HUVECs before exposing to HGHF.

Glutamine consumption in the substrate was figured out using the Glutamine Assay Kit (catalog no. EGLN-100, Bioassay Systems, Hayward, USA). The procedure involved incubating aliquots of the substrate through the specific enzyme mixes for 40 min at ordinary temperature in darkness. Following this incubation, absorbance was measured at 565 nm using a microplate spectrophotometer (Thermo Fisher Scientific). To determine the levels of intracellular glutamate and glutamine metabolites among HUVECs, gas chromatography–mass spectrometry (GC-MS) analysis was performed [53]. It was tested by Shanghai ProfLeader Technology Co. Ltd. (Shanghai, China). and the experimental setup was replicated at least 3 times.

### Molecular docking

SalB is semi-flexibly docked with the core target to explore its interactions and predict binding patterns and affinity. The crystal structure of human receptor with similar structure and high resolution was selected. ChemBio Draw Ultra 14.0 was used to draw the structure of SalB, and it was saved in Mol2 format. The Autodock Vina program docking was run to verify the combination of SalB with the core target. Potential pharmacoactive substances were screened according to binding energy below −5.0 kcal/mol, and the binding patterns were visualized by Discovery Studio software [54].

### Migration assays

For the cell scratch assay, 2 × 10^5^ cells/well were fostered into a 6-well plate and grew until confluent. The monolayer was then subjected to a scratch wound using a sterile micropipette tip followed by washing through the substrate to remove nonadherent cells. The HUVECs were then cultivated within HGHF substrate or Ctrl medium treated with vehicle, 10 μM SalB, 20 μM SalB, or 2 mM α-KG plus 0.2 mM NEAAs for 48 h. To evaluate cell migration, one field per well was captured at regular intervals during wound healing and the quantity of cells that migrated in each group was quantified using a microscope (Leica DMI6000B, Solms, Germany). For each analysis, 3 independent experiments were conducted to ensure the reliability and reproducibility of the results.

### Capillary-like tube formation assay

Growth factor-reduced Matrigel (BD Biosciences, USA) was applied to 96-well plates and permitted to solidify for 15 min at 37 °C. Subsequently, 2 × 10^4^ HUVECs per well were seeded in triplicate for each group onto the solidified Matrigel and subjected to one of the following treatments: vehicle control, 10 μM SalB, 20 μM SalB, or a combination of 2 mM α-KG and 0.2 mM NEAAs. After incubation for 48 h under 95% air and 5% CO_2_ at 37 °C, tube formation by cells was examined microscopically. The total number of branching points per field with the cumulative tube length was calculated through Image-Pro Plus 6 software (Media Cybernetics, USA).

### Aortic capillary sprouting assay

Multiple thoracic aorta fragments (approximately 500 μm in length, with more than 3 fragments each aorta) were meticulously anatomized from 2 to 3 mice in each experimental group and embedded within Matrigel according to established protocols [17]. The explants were monitored under a microscope for up to 4 d. To evaluate the extent of ex vivo angiogenesis, the region of capillary sprouting was quantified through Image-Pro Plus 6 software.

### MI/R injury model

Twelve-week-old male db/db mice or GLS^ΔEC^ mice and their respective control littermates underwent MI/R impairment by LAD ligation, according to previously described methods [55]. Specifically, when the mice were anesthetized, chest cavity was opened at the fourth intercostal space under mechanical ventilation. The LAD coronary artery was ligated for 30 min with an 8-0 silk suture. After removing the silk suture, after closing thoracic incision, the mice were placed on a heated blanket until recovery from anesthesia. The sham group experienced cardiac exposure and thoracotomy without undergoing LAD ligation. Post-surgery, mice were segregated into 5 groups as per the experimental protocol: sham, MI/R (30 min of ischemia/7-d reperfusion with vehicle treatment), MI/R + SalB low dose (40 mg/kg/d intraperitoneal injection), MI/R + SalB high dose (80 mg/kg/d intraperitoneal injection), and MI/R + α-KG (10 mg/kg/d intraperitoneal injection). Group allocation was based on the specific requirements of the experiments. At the conclusion of the study, when the mice had reached 13 weeks of age, their hearts were harvested for analysis. All subsequent analyses were conducted in a blinded manner.

### Target gene up-regulation

Ad-EV and Ad-GLS1 were formulated by Hanbio Biotechnology Ltd. (Shanghai, China). The viral titer for these adenoviruses was approximately 1.2 × 10^10^ plaque-forming units per milliliter (PFU/ml). To perform intramyocardial injections of the adenoviruses, we adhered to the methodology previously detailed: After the mice were anesthetized using 3% isoflurane, tracheal intubation was performed. Throughout the subsequent surgical procedure, the anesthetized state was maintained with 2% isoflurane [55]. Upon exposure of the hearts, the adenovirus was injected into the ventricular free wall left at 4 distinct positions, with a volume of 10 μl administered at each site using a Hamilton 705RN syringe equipped with a 50-μl needle (Hamilton, USA) [56].

### Immunofluorescence analysis of cardiac tissue

Immunofluorescence staining was employed to rate the degree of vascular EC and newly formed capillaries during angiogenesis in cardiac tissue. Briefly, heart samples were collected on day 7 post-MI/R, immobilized within 3.5% paraformaldehyde, and placed in the optimum cutting temperature (OCT) compound following dehydration in a 30% sucrose solution. Serial slides, 10 μm thick, were incubated all night at 4 °C with primary antibodies: anti-CD31 (ab28364, Abcam; diluted 1:300) or anti-α-SMA (ab5694, Abcam; diluted 1:300). Following PBS washes, the slides were brooded through one HRP-conjugated goat anti-rabbit secondary antibody (ab97075, Abcam; diluted 1:200) for 1 h at 37 °C. The antigen–antibody complexes were visualized using diaminobenzidine tetrahydrochloride, and the slides stained were checked through an optical microscope. Fluorescent images were captured through a Leica DMI6000B fluorescence microscope (Leica Microsystems, Germany). To quantify differences in blood vessel numbers among the groups, 5 random fields from each section within the myocardial infarct border zone were construed through Image-Pro Plus 6 software.

### Echocardiography

Echocardiography was conducted on mice utilizing a VEVO 2100 Imaging System (Fujifilm Visual Sonics) to document the left ventricular systolic and diastolic motion profiles. Such a procedure was carried out under anesthesia, with continuous electrocardiogram (ECG) monitoring to track heart rate. M-mode echocardiographic images were acquired to measure the internal diameter of LVIDs and LVIDd. Computer algorithms were applied to calculate the left ventricular end-diastolic volume (LVEDV) and end-systolic volume (LVESV). LVEF and LVFS were then determined using these formulas: LVEF (%) = [(LVEDV − LVESV)/LVEDV] × 100, LVFS (%) = [(LVIDd − LVIDs)/LVIDd] × 100. All echocardiographic data were analyzed using the dedicated software provided with the VEVO 2100 system [56].

### Mouse skin wound model and treatment

The research received approvals from the Committee on Care and Use of Laboratory Animals at the Fourth Military Medical University to ensure ethical treatment of animals. Male mice, at the age of 6 to 8 weeks and weighing 22 ± 2 g, were anesthetized prior to the creation of an excisional full-thickness skin wound model on their dorsum using a 5-mm biopsy punch (Integra Miltex, York, PA, USA). Mice were randomly assigned to receive subcutaneous injections of vehicle, SalB (80 mg/kg/d), SalB (80 mg/kg/d) plus BPTES (5 mg/kg/d), or α-KG (10 mg/kg/d) at 4 injection sites around the wounds. The doses were determined according to a published study [22,57,58]. After 14 d, the mice were sacrificed, and blinding was used in subsequent analyses. Skin samples were harvested for further analysis using immunofluorescence techniques [59].

### Evaluation of wound closure and blood vessel regeneration

The wound size photographs were obtained on days 0, 3, 7, and 14 after surgery to assess the progress of the healing process in each group. The size of the wounds was measured using one caliper ruler, with the wound region calculated through Image-Pro Plus 6 software. Wound closure was estimated by the following formula: Wound closure (%) = [(Wound area of day 0 − Wound area of day *n*)/Wound area of day 0] ×100. The formation of neovessels was examined by observing and capturing images of the underside of the skin on day 14 post-wounding.

### Statistical analysis

All results are presented as mean ± standard error (SEM). The statistical significance was determined through the 2-tailed, unpaired Student’s *t* test; the contrast between multiple groups was implemented by least significant difference (LSD) assay after the one-way analysis of variance (ANOVA) analysis showed no difference in variance; the difference in variance was evaluated by Games–Howell assay. Post hoc assays were implemented only if *F* in ANOVA achieved *P* < 0.05. Statistical significance was considered as *P* < 0.05. The statistical analyses were conducted through GraphPad Prism (v 8.0) (GraphPad Software, USA).

### Materials

SalB (purity > 98%) was supplied by Honson Biotechnology Co. Ltd. (China) (catalog no. w2740010). Bicinchoninic acid (BCA) protein assay kit was produced by Sangon Biotech Co. Ltd. (China) (catalog no. c503061). BPTES was obtained from Sigma-Aldrich Co. Ltd. (Missouri, USA) (catalog no. SML0601). PBS was supplied by Sigma-Aldrich Co. Ltd. (Missouri, USA) (catalog no. 806552). α-KG was offered by Sigma-Aldrich Co. Ltd. (Missouri, USA) (catalog no. 349631). Glutaminase Assay Kit was provided by Biomedical Research Service Center (Buffalo, USA) (catalog no. E-133). Glutamine Assay Kit was obtained from Bioassay Systems (Hayward, CA, USA).

## Ethical Approval

Our research was implemented according to the principles Helsinki Declaration and Basel Declaration. The collected human skin samples received approvals from the Medical Ethics Committee of the Xijing Hospital, Fourth Military Medical University (approval no. KY20233600-1). Before gathering tissue samples, informed consent was supplied by each patient. The experiments involving animals were conducted in compliance with the National Institutes of Health Guidelines for the Use of Laboratory Animals and were approved by the Committee on the Care and Use of Laboratory Animals of the Fourth Military Medical University (approval no. KY20230110).

## Supplementary Material

20230426-1

## Data Availability

Data are included in the paper or uploaded as Supplementary Materials.
