# Erratum to “Defective Endothelial Glutaminolysis Contributes to Impaired Angiogenesis and Poor Ischemic Tissue Repair in Diabetes”

**DOI:** 10.34133/research.0952

**Published:** 2025-11-13

**Authors:** Meina Zhao, Jiaheng Zhou, Yang Hu, Xinpei Wang, Jiong An, Meijie Liu, Pengfei Zhang, Xing Zhang, Jingwen Wang, Xing Jin, Miaomiao Xi, Jia Li

**Affiliations:** ^1^ Key Laboratory of Aerospace Medicine of Ministry of Education, School of Aerospace Medicine, Key Laboratory of Preventive Medicine of Ministry of Education, Fourth Military Medical University, Xi’an, Shaanxi 710032, China.; ^2^ Department of Pharmacy, Xijing Hospital, Fourth Military Medical University, Xi’an, Shaanxi 710032, China.; ^3^ Department of Cardiology, General Hospital of Central Theater Command, Wuhan, Hubei 430061, China.; ^4^ Senior Department of Ophthalmology, the Third Medical Center of PLA General Hospital, Beijing 100853, China.; ^5^ TANK Medicinal Biology Institute of Xi’an, Xi’an, Shaanxi 710032, China.; ^6^ Ministry of Education Key Lab of Hazard Assessment and Control in Special Operational Environment, Fourth Military Medical University, Xi’an, Shaanxi 712000, China.

The authors have identified an error in the Research Article entitled “Defective Endothelial Glutaminolysis Contributes to Impaired Angiogenesis and Poor Ischemic Tissue Repair in Diabetes” [1].

In Fig. 1B: Following a thorough review of the manuscript, and in the interest of maintaining the integrity of the published work, we have provided the original, traceable blot images for Figure 1B to facilitate accurate correction and verification.

In Fig. 4I: The same image was inadvertently reused in both the HGHF+SalB 20 μM group and the HGHF+α-KG+NEAA group. This occurred due to an operational oversight involving repeated copying of image files, confusion between uploaded versions, and time-sensitive upload errors. We sincerely apologize for this unintentional error, which was purely technical in nature and does not reflect any intentional deviation. This incident does not affect the core data or analytical framework of the study, and the primary conclusions remain fully supported by the validated experimental findings.

The corrected figures are below, and the original HTML and PDF have been updated to reflect this change.

**Fig. 1.**
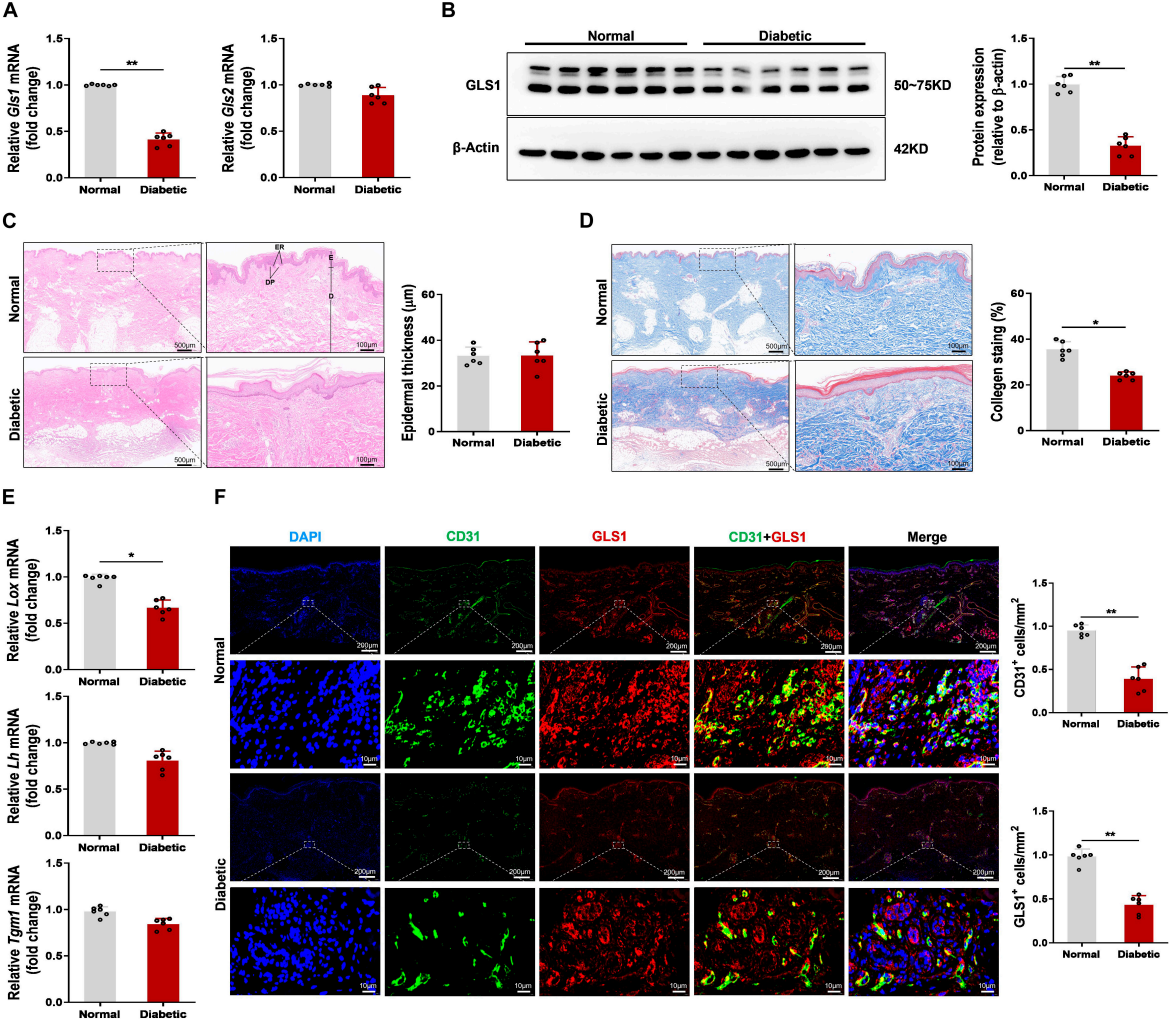
Glutaminase 1 (GLS1) expression was decreased and reduced colocalization with the endothelial cell (EC) marker platelet-endothelial cell adhesion molecule-1 (CD31) in skin wound healing of patients with diabetes after tissue injury. (A) Relative mRNA expression of *GLS1* and *GLS2* in human skin wound from diabetic and nondiabetic patients at day 3 after acute trauma. The data were standardized to the reference gene *18s rRNA*. (B) Representative blot images and quantitative analysis of GLS1 expression in diabetic and nondiabetic patients at day 3 after acute trauma. (C) Representative hematoxylin and eosin (H&E) images in human skin wound from diabetic and nondiabetic patients at day 3 after acute trauma. The full-thickness skin showed the epidermis (E) and the dermis (D). Arrows indicated the epidermal ridges (ERs) and the dermal papillae (DP). Scale bar, 500 μm (left) or 100 μm (right). (D) RepresentativeMasson trichrome staining in human skin wound from diabetic and nondiabetic patients at day 3 after acute trauma. Dark blue staining indicates abundant collagen. Scale bar, 500 μm (left) or 100 μm (right). (E) Relative mRNA expression of *lysyl oxidases (Lox), lysyl hydroxylases (Lh)*, and *transglutaminase 1 (Tgml)* in human skin wound from diabetic and nondiabetic patients at day 3 after acute trauma. The data were standardized to the reference gene *18s rRNA*. (F) Representative immunofluorescence images showed that GLS1 (red) colocalized with the EC marker CD31 (green) in human skin wound sections from diabetic and nondiabetic patients at day 3 after acute trauma. Scale bar, 200 μm (top) or 10 μm (bottom). *n* = 6/group, 6 diabetic patients and 6 matched nondiabetic patients were construed. Values are expressed as mean ± SEM. *P < 0.05; **P < 0.01.

**Fig. 4.**
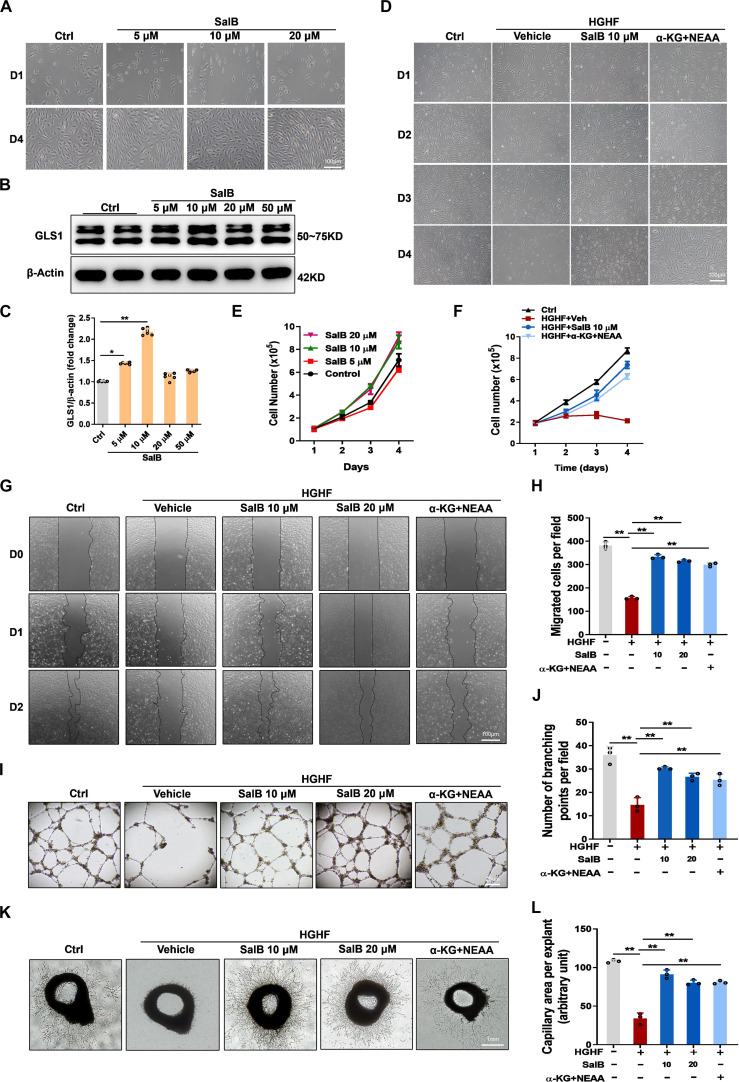
SalB treatment or α-ketoglutarate (α-KG) plus nonessential amino acids (NEAAs) supplementation rescued EC proliferation and migration in the HGHF condition. HUVECs were exposed to HGHF medium or Ctrl medium treated with vehicle (Veh), SalB (5, 10, and 20 μM) or cell-permeable α-KG (2 mM) plus NEAAs (0.2 mM). (A and E) Phase contrast images and growth curves of HUVECs following 4-d culture within Ctrl medium with the increasing concentrations of SalB (5, 10, and 20 μM). Scale bar, 100 μm. (B and C) Representative blot images and quantitative analysis of GLS1. (D and F) Phase contrast images (original magnification, ×40) and growth curves of HUVECs during 4 d of different treatments. Scale bar, 100 μm. (G and H) Representative images (original magnification, ×40) of cell scratch test and quantitative analysis of HUVECs during 48 h of different treatments. Scale bar, 100 μm. (I and J) Tube-forming capacity of HUVECs after 48 h of different treatments. Representative images (original magnification, ×10) describing the forming of capillary-like tube constructions by HUVECs on Matrigel. Each tube was photographed, and the number of branching was quantified by using ImageJ. Scale bar, 25 μm. (K and L) Capillary sprouting from mouse aortic explants with different treatments for 4 d. Each explant was photographed, and the area of capillary outgrowth was quantified by using ImageJ. Scale bar, 1 mm. Values are represented as mean ± SEM from 3 independent experiments. *P < 0.05; **P < 0.01.
