# Supplementary material for: Defective Endothelial Glutaminolysis Contributes to Impaired Angiogenesis and Poor Ischemic Tissue Repair in Diabetes
Source: Research (Wash D C). 2025 May 22;8:0706. doi: 10.34133/research.0706 (PMC12095913; doi:10.34133/research.0706)
Supplement: Supplementary 1 — Figs. S1 to S6 Tables S1 to S2 [file research.0706.f1.zip › Supplementary Table 1.docx]

**Supplementary Table 1.** Information about patient samples used.

| Number of patients | BMI, kg/m^2^ | HbA_1c_, % (mmol/mol) | Case # | Age(years) | Sex | DM Type |
| --- | --- | --- | --- | --- | --- | --- |
| 6 | 30.2±0.8* | 7.9±0.3* (62±3) | DM-1 | 58 | M | IDDM |
|  |  |  | DM-2 | 42 | M | NIDDM |
|  |  |  | DM-3 | 63 | M | NIDDM |
|  |  |  | DM-4 | 59 | M | IDDM |
|  |  |  | DM-5 | 46 | F | IDDM |
|  |  |  | DM-6 | 49 | M | NIDDM |
| 6 | 23.1±0.7 | 5.1±0.1 (36±1) | N-1 | 60 | F | / |
|  |  |  | N-2 | 40 | M | / |
|  |  |  | N-3 | 78 | F | / |
|  |  |  | N-4 | 34 | M | / |
|  |  |  | N-5 | 39 | M | / |
|  |  |  | N-6 | 46 | M | / |

*, *P* < 0.05 *VS* non-diabetic patients. M, male; F, female; IDDM, type 1 diabetes; NIDDM, type 2 diabetes.
