# Supplementary material for: Defective Endothelial Glutaminolysis Contributes to Impaired Angiogenesis and Poor Ischemic Tissue Repair in Diabetes
Source: Research (Wash D C). 2025 May 22;8:0706. doi: 10.34133/research.0706 (PMC12095913; doi:10.34133/research.0706)
Supplement: Supplementary 1 — Figs. S1 to S6 Tables S1 to S2 [file research.0706.f1.zip › Supplementary Table 2.docx]

**Supplementary Table 2.** Sequences of primers used for qRT-PCR.

| Genus | mRNA | Forward | Reverse |
| --- | --- | --- | --- |
| mu | *Glut1* | GACAACGCCCGGCGTCTGACTGG | CAATTGGCAGAAGGCCGACGGTC |
| mu | *Pfkfb3* | TATGAAGCCAGCTACCAGCC | TCTGGATGTGGTCCTGCAC |
| mu | *Pkm2* | GGAGGCTGTTCGCATGCAGCACC | GGCGGAGTTCCTCGAATAGCTGC |
| mu | *Hk2* | GGAGCTACCACACACCCTACAGC | GCACACGGAAGTTGGTTCCTCC |
| mu | *Gls* | GCACTACACTTTGGACACCA | TAGCAACCCGTCGAGATT |
| mu | *Glud1* | CCCAACTTCTTCAAGATGGTGG | AGAGGCTCAACACATGGTTGC |
| hu | *Glut1* | AAGGTGATCGAGGAGTTCTACA | ATGCCCCCAACAGAAAAGATG |
| hu | *Pfkfb3* | AAGCAGTACAGCTCCTACAACT | CTTCTTTCGCCAGGTAGCTTT |
| hu | *Pkm2* | TGAGGCAGAGGCTGCCATCTACCA | TGCCAGACTTGGTGAGGACGATTATGG |
| hu | *Hk2* | GGGACAATGGATGCCTAGATG | GTTACGGACAATCTCACCCAG |
| hu | *Gls* | TCTACAGGATTGCGAACGTCT | CTTTGTCTAGCATGACACCATCT |
| hu | *Glud1* | AGGAATGACACCAGGGTTTG | TCAGACTCACCAACAGCAATAC |
| hu | *Lox* | ACCAAGGGACATCAGATTTCTT | AGCAGGTCATAGTGGCTAAAC |
| hu | *Lh* | TGTCCTCAATCTCGACACTACTA | GATAACTTTGGTCTCCCTCTTACC |
| hu | *Tgm1* | GGATCTGATGGTCTCTGTGATG | CTTCCTTCTTGGTCTCCTTGAA |
| mu&hu | *18s* | GTAACCCGTTGAACCCCATT | CCATCCAATCGGTAGTAGCG |
