# Supplementary figures and images for: Defective Endothelial Glutaminolysis Contributes to Impaired Angiogenesis and Poor Ischemic Tissue Repair in Diabetes
Source: Research (Wash D C). 2025 May 22;8:0706. doi: 10.34133/research.0706 (PMC12095913; doi:10.34133/research.0706)

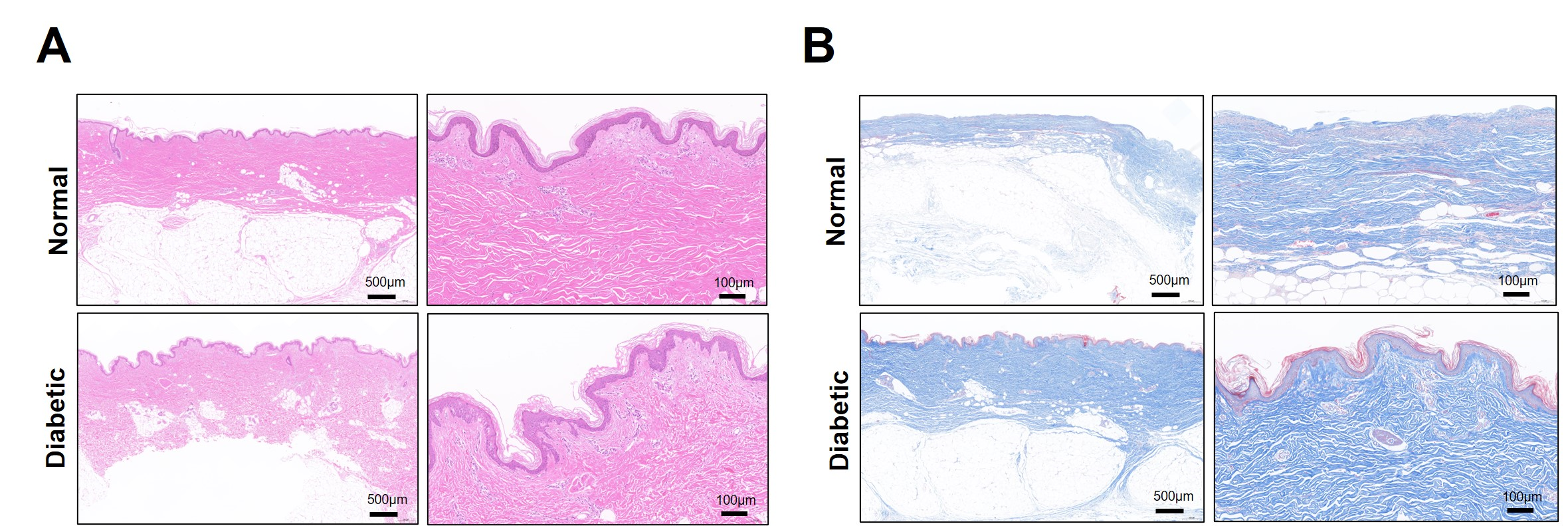

Supplement: Supplementary 1 — Figs. S1 to S6 Tables S1 to S2 [file research.0706.f1.zip › SF-1.tiff]

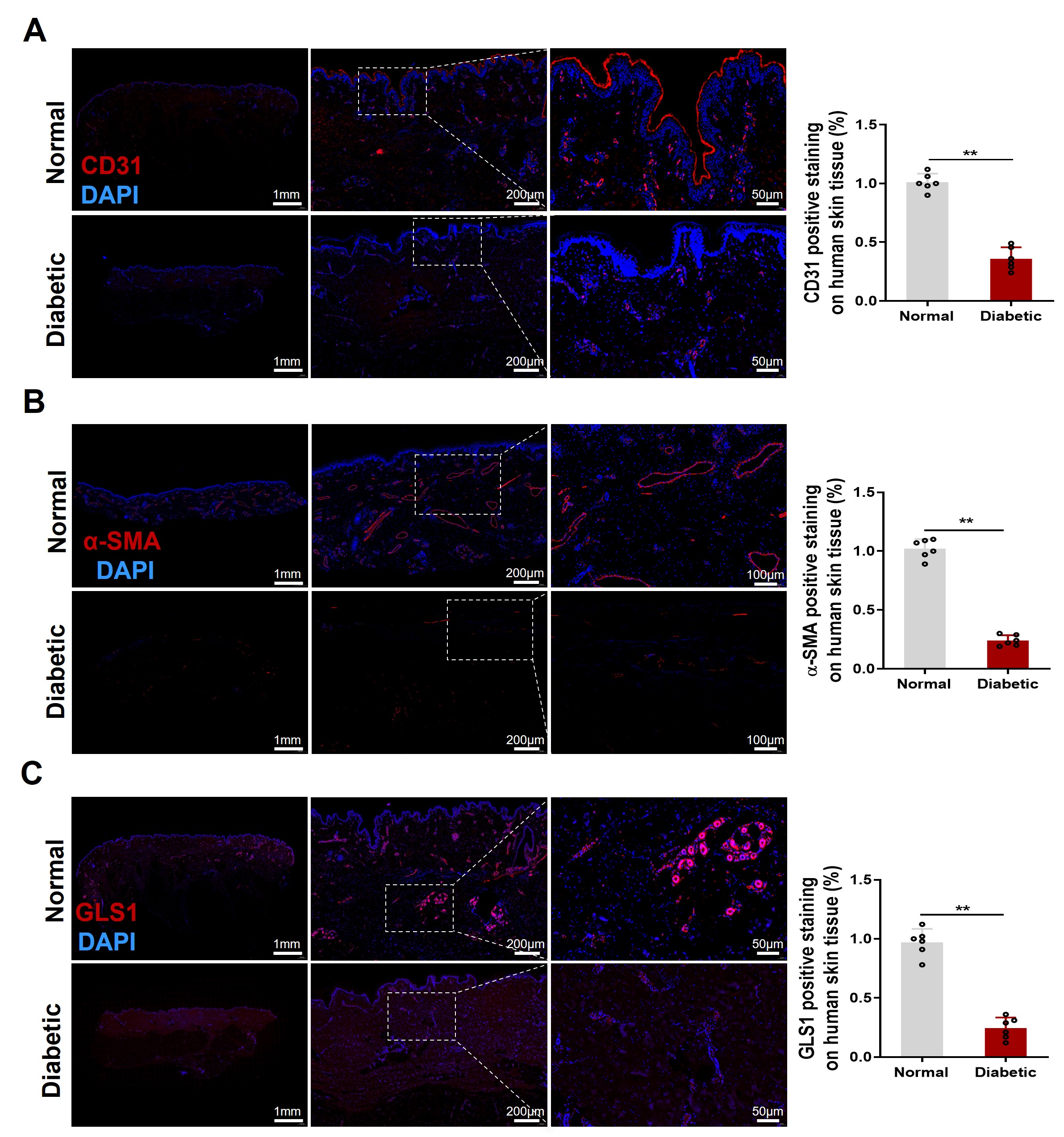

Supplement: Supplementary 1 — Figs. S1 to S6 Tables S1 to S2 [file research.0706.f1.zip › SF-2.tiff]

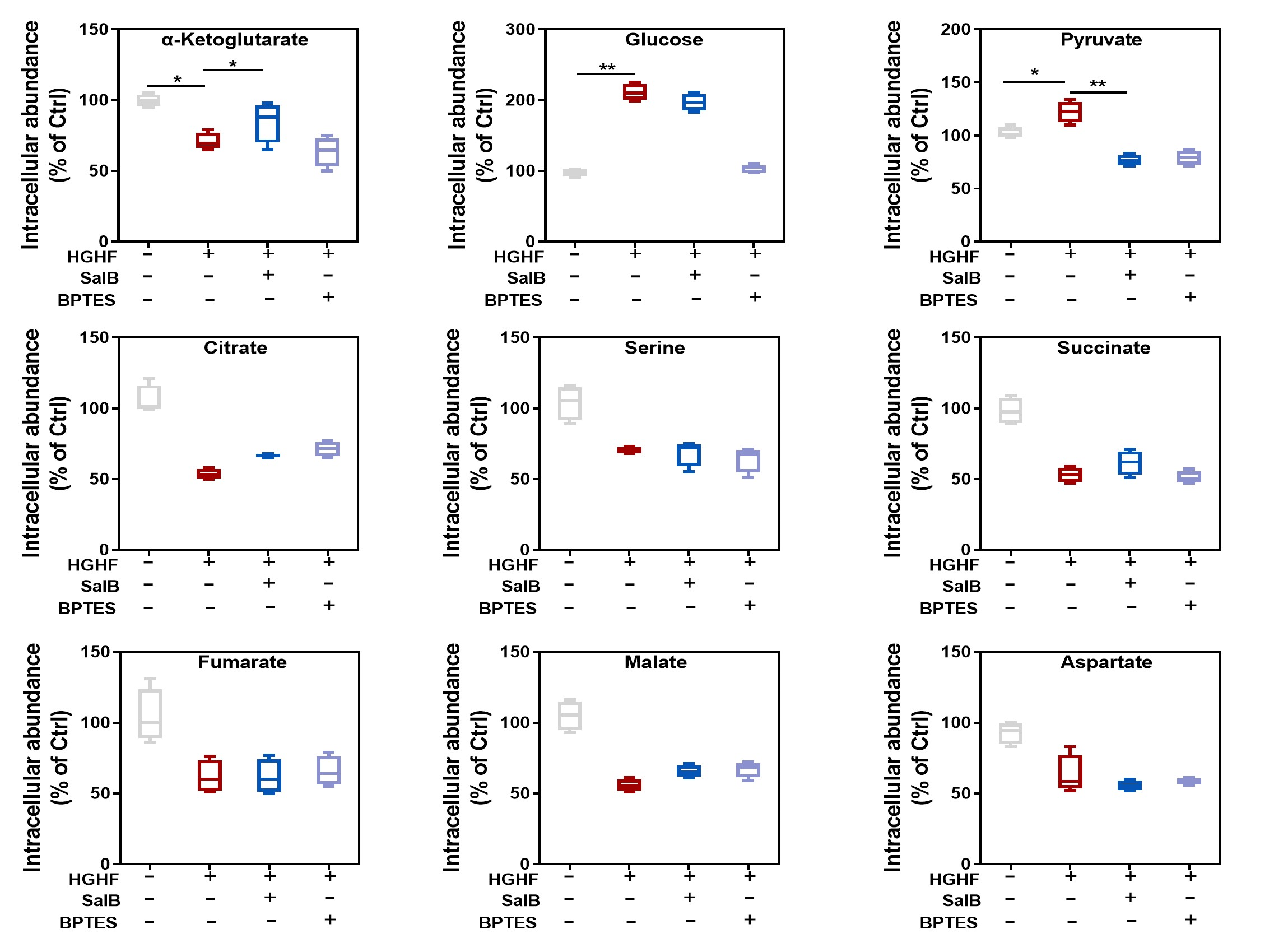

Supplement: Supplementary 1 — Figs. S1 to S6 Tables S1 to S2 [file research.0706.f1.zip › SF-3.tiff]

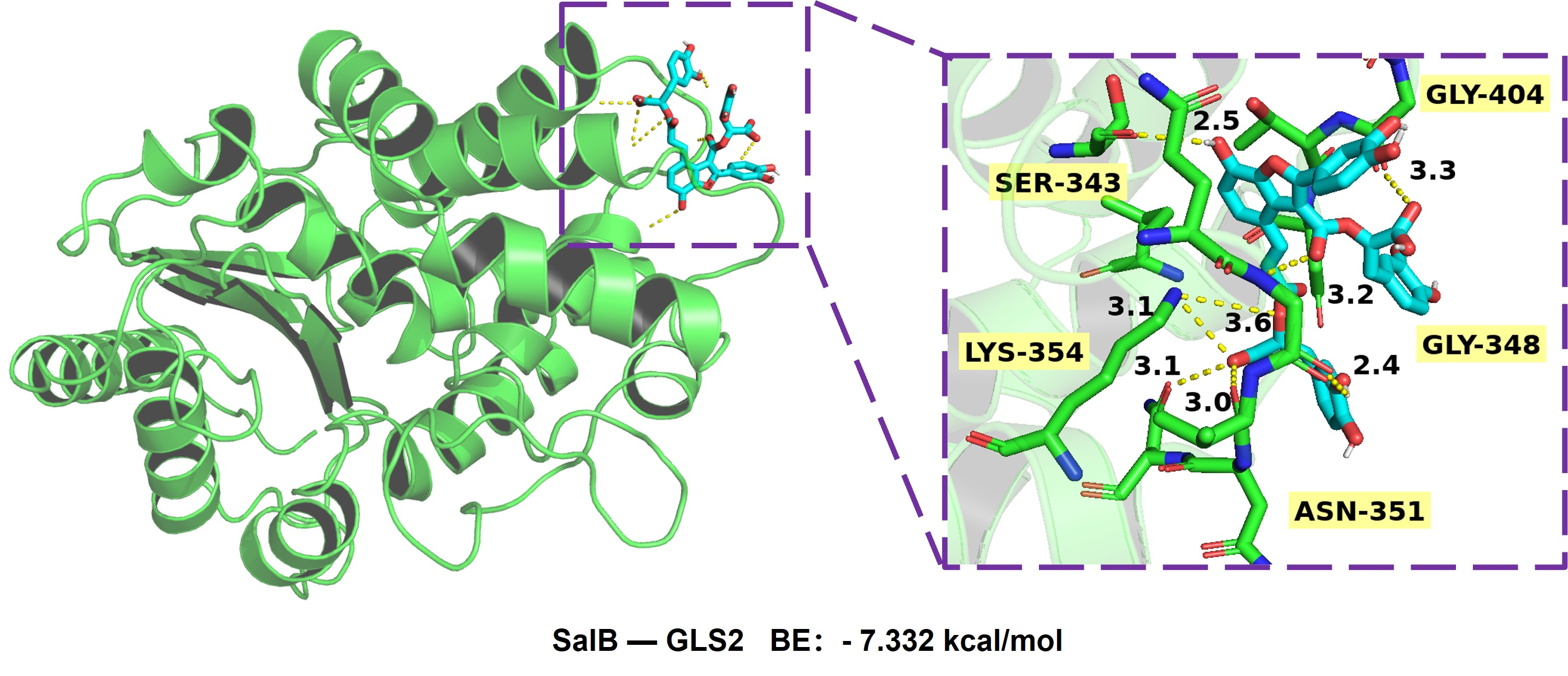

Supplement: Supplementary 1 — Figs. S1 to S6 Tables S1 to S2 [file research.0706.f1.zip › SF-4.tiff]

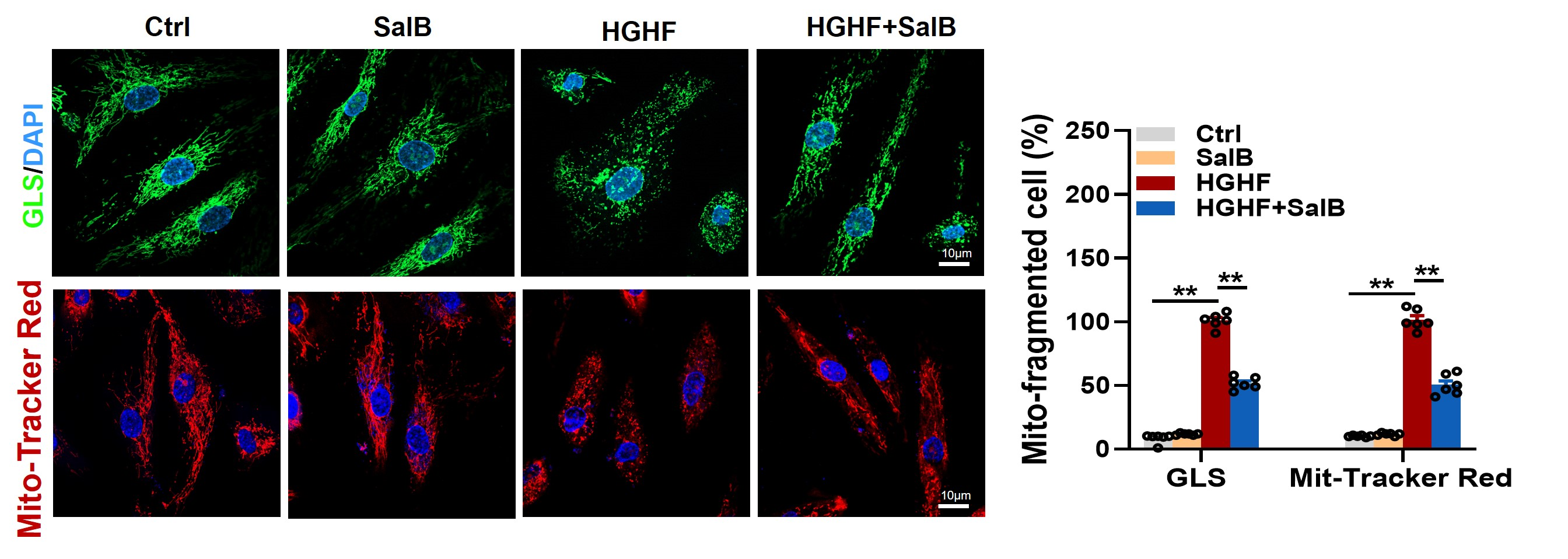

Supplement: Supplementary 1 — Figs. S1 to S6 Tables S1 to S2 [file research.0706.f1.zip › SF-5.tiff]

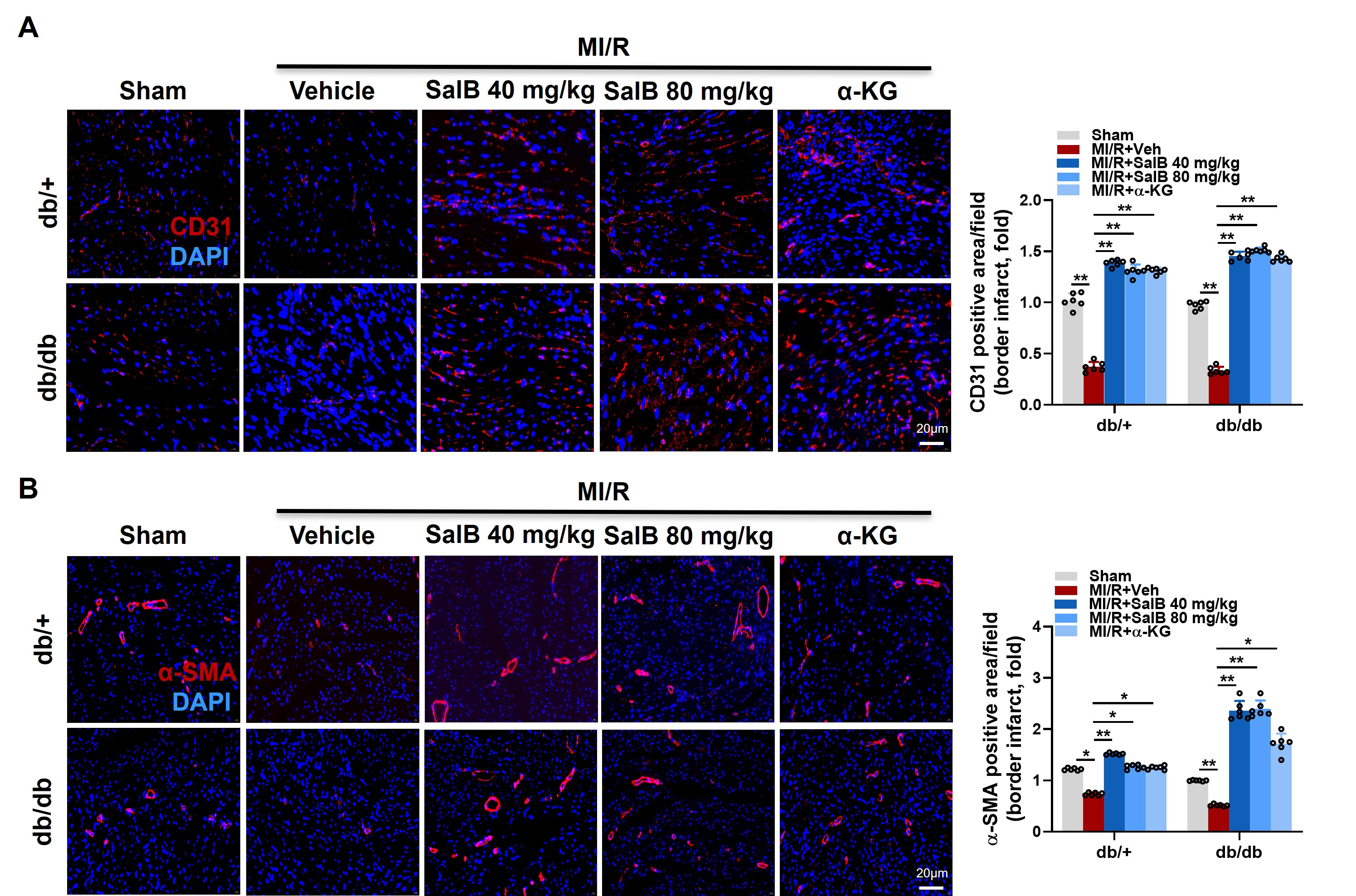

Supplement: Supplementary 1 — Figs. S1 to S6 Tables S1 to S2 [file research.0706.f1.zip › SF-6.tiff]
